# Assessment of future meteorological droughts in Iran

**DOI:** 10.1038/s41598-026-65752-6

**Published:** 2026-08-28

**Authors:** Babak Ghazi, Hossein Salehi, Marco Turco, Amir AghaKouchak

**Affiliations:** 1 Department of Geoscience, University of Nevada, Las Vegas, Las Vegas, USA; 2 Department of Physics, University of Trento, Trento, Italy; 3 Regional Atmospheric Modelling Group, Regional Campus of International Excellence Campus Mare Nostrum, University of Murcia, 30100 Murcia, Spain; 4 Department of Civil and Environmental Engineering, University of California, Irvine, Irvine, CA USA; 5 Department of Earth System Science, University of California, Irvine, Irvine, CA USA; 6 United Nations University Institute for Water, Environment and Health, Hamilton, ON Canada; 7 Center Agriculture, Food and Environment (C3A), University of Trento, Trento, Italy

**Keywords:** Climate change, Droughts, NEX-GDDP, SPI, SPEI, CMIP6, Iran, Climate sciences, Hydrology, Water resources

## Abstract

Climate change amplifies the risk of hydroclimatic extremes, posing significant challenges for arid and semi-arid regions vulnerable to temperature and precipitation variability. This study projects future meteorological drought in Iran under different climate change scenarios, focusing on two key objectives: (1) evaluating the performance of the NASA Earth Exchange Global Daily Downscaled Projections (NEX-GDDP) from the Coupled Model Intercomparison Project Phase 6 (CMIP6), and (2) quantifying future drought conditions using the Standardized Precipitation Index (SPI) and the Standardized Precipitation Evapotranspiration Index (SPEI). We employ a multi-model ensemble of 25 bias-corrected and downscaled General Circulation Models (GCMs) under four Shared Socioeconomic Pathways (SSP1–2.6, SSP2–4.5, SSP3–7.0, and SSP5–8.5) to project variations in temperature, precipitation, and meteorological drought occurrence in Iran for 2071–2100. The novelty of this study lies in integrating an Iran-specific evaluation of NEX-GDDP-CMIP6 with multi-timescale SPI–SPEI comparison and quantitative attribution of projected drought changes to precipitation and potential evapotranspiration (PET). Results reveal pronounced warming, especially under SSP5–8.5, exceeding 5 °C in many parts of the country by 2100. Although precipitation projections show slight increases overall, notably under SSP3–7.0, substantial regional variability persists. Projections indicate that meteorological droughts are expected to become more frequent and severe in the northwestern and southeastern parts of Iran, with SPEI indicating greater drought intensity and duration due to temperature-driven increases in PET. These findings highlight the need for climate-resilient water management and robust adaptation policies to reduce the impacts of future droughts.

**Supplementary Information:**

The online version contains supplementary material available at https://doi.org/10.1038/s41598-026-65752-6.

## Introduction

Climate change and variability are among the most critical environmental challenges, particularly in arid and semi-arid regions, where both ecosystems and communities are highly vulnerable to fluctuations in water availability^1^. Variations in climatic parameters, such as temperature and precipitation, can intensify the occurrence and severity of hydroclimatic extremes^2,3^. Droughts, in particular, are a complex phenomenon with serious consequences for the agricultural and hydrological sectors^4^. Previous studies indicate that many of the world’s dry regions are projected to face even more severe water stress^5,6^.

As a largely semi-arid country in the Middle East, Iran has felt the intensifying impacts of climate change and hydroclimatic variability, compounded by unsustainable growth and water management practices^7^. Observations over recent decades demonstrate a clear warming trend and persistent droughts, floods, and shrinking water bodies^8–10^. Multiple studies report a rise in drought frequency, severity, and duration, threatening already strained water resources^11,12^. While many of the observed impacts in Iran are influenced by human activities, a phenomenon often referred to as anthropogenic drought^13^, meteorological drought still plays a significant role in water availability^14^. This underscores the importance of evaluating future climate impacts on drought occurrences.

The majority of prior research projecting future droughts in Iran utilized climate outputs from Coupled Model Intercomparison Project Phase 3 (CMIP3) and Coupled Model Intercomparison Project Phase 5 (CMIP5) models and outdated Representative Concentration Pathway (RCP) scenarios^10,15–19^. Here, we utilize the Coupled Model Intercomparison Project Phase 6 (CMIP6) simulations, which represent substantial advancements over the previous phase. CMIP6 models incorporate enhanced dynamic processes, finer spatial resolutions, and use new Shared Socioeconomic Pathways (SSP) that offer improved representation of future climate forcing and uncertainties^20^. This state-of-the-art set of models significantly increases the reliability and scope of future drought projections. Therefore, enhancing the current understanding of future drought conditions in Iran using high-resolution CMIP6 simulations under various SSP scenarios is essential.

Most CMIP6-based drought projection studies for Iran focus on local case studies or on a few representative clusters rather than providing full national coverage^21–23^. For example, one study grouped synoptic stations across the country into five clusters, represented by Ahvaz, Chalus, Kaboudarahang, Minab, and Tabas, which may not fully capture the entire region’s climate and drought variability^24^. Another examines drought severity and duration using only three CMIP6 GCMs (CanESM5, GFDL-ESM4, IPSL-CM6A-LR) under two SSPs (SSP1–2.6 and SSP5–8.5), applying the Standardized Precipitation Index (SPI) and the Standardized Precipitation Evapotranspiration Index (SPEI)^25^. While informative, this limited model ensemble, together with bias correction applied to coarse-resolution native GCM outputs, constrains robustness and spatial detail in heterogeneous climatic regions. In addition, the use of the Thornthwaite method for potential evapotranspiration (PET) may overestimate drought projections in arid regions such as Iran^25^. In contrast, our study uses the bias-corrected and statistically downscaled NASA Earth Exchange Global Daily Downscaled Projections (NEX-GDDP) CMIP6 dataset at 0.25° spatial resolution, which provides finer spatial detail than native GCM outputs and allows a more comprehensive assessment of Iran’s regional climate variability. Nevertheless, this resolution does not fully resolve sharp orographic gradients, and its performance in complex terrain is therefore evaluated separately in this study. In addition to the above-mentioned national-scale drought studies, broader studies have assessed the future of droughts based on CMIP6 simulations globally^26–30^ or across the Middle East^31,32^. Although these large-scale assessments provide valuable regional and global context, drought dynamics are strongly influenced by local climatic, topographic, and hydrological conditions that vary considerably among geographic domains^33^. A country-specific analysis is therefore necessary to assess whether downscaled climate projections adequately represent Iran’s highly heterogeneous climate and to determine how the mechanisms of future drought change vary across the country.

Despite recent advances, several important gaps remain in previous drought projections for Iran. Most studies have focused primarily on the projected magnitude, frequency, or duration of drought, while less attention has been paid to assessing the capabilities and limitations of the underlying downscaled climate dataset before it is used for drought projection. In particular, previous studies have not jointly evaluated whether a large NEX-GDDP-CMIP6 ensemble reproduces the distributional characteristics of observed temperature and precipitation across Iran, how model performance varies with elevation, whether projected changes are statistically robust across individual GCMs, and whether the adopted drought indices reproduce historically documented drought variability. Furthermore, although previous studies have recognized the importance of warming-driven increases in PET, the separate contributions of precipitation and PET to projected SPEI changes have not been comprehensively quantified across Iran under four SSP scenarios and multiple drought-accumulation timescales.

To address these gaps, this study develops an integrated evaluation–projection–attribution framework using 25 bias-corrected and statistically downscaled NEX-GDDP-CMIP6 models at 0.25° spatial resolution under SSP1–2.6, SSP2–4.5, SSP3–7.0, and SSP5–8.5 scenarios. First, the historical performance of the ensemble is evaluated against observations from 51 meteorological stations by examining the central tendency, variability, distributional shape, and tails of temperature and precipitation distributions. The evaluation is further extended through an elevation-based comparison of lowland, mid-altitude, high-elevation, and high-mountain stations.Additionally, we assessed seasonal inter-model spread and examined spatial agreement and trend significance across individual GCMs. We also verified whether the observed SPI and SPEI time series reproduce the temporal evolution and persistence of historically documented drought conditions. These analyses provide a more complete assessment of both the suitability and the remaining limitations of NEX-GDDP-CMIP6 for climate-change and drought applications in Iran, particularly in complex high-elevation regions where orographic precipitation may be spatially smoothed.

Second, the study moves beyond describing future drought patterns by investigating the physical and climatic mechanisms that produce them. Projected changes in temperature and precipitation are interpreted in relation to atmospheric circulation, moisture transport, topographic controls, seasonal variability, and the thermodynamic effects of warming. Drought probability, intensity, and duration are then assessed using both SPI and SPEI at 1-, 6-, and 12-month accumulation timescales. The agreement between precipitation-based and PET-sensitive drought classifications is quantified using Cohen’s Kappa, allowing us to determine how the correspondence between the two indices changes as warming intensifies. Most importantly, projected changes in SPEI are decomposed into separate precipitation- and PET-related contributions. This attribution distinguishes drought changes caused by precipitation variability from those amplified by temperature-driven increases in PET and explains why SPEI-based drought may intensify even in areas or scenarios where precipitation remains stable or increases slightly.

Third, the analysis evaluates how future drought characteristics vary across SSP scenarios, accumulation timescales, seasons, climatic regions, and different stages of the twenty-first century. The assessment of both mid-century and late-century periods helps identify when warming-driven drought intensification begins to emerge rather than considering only end-of-century conditions. The spatial analyses further distinguish regions where projected drought is primarily associated with precipitation variability from regions, particularly the central and southern arid areas, where increasing PET is the dominant driver of declining climatic water balance.

Taken together, the novelty of this study lies not in the use of CMIP6 projections, SPI, or SPEI individually, but in integrating dataset evaluation, uncertainty and robustness assessment, historical drought verification, multi-timescale SPI–SPEI comparison, quantitative precipitation–PET attribution, and process-based interpretation within one nationally consistent framework. To our knowledge, these components have not previously been combined in a single assessment of future meteorological drought across Iran. This framework provides new information not only on where and when drought conditions are projected to intensify, but also on why they change and how confidently the resulting patterns can be interpreted.

Accordingly, the objectives of this study are to: (1) evaluate the capability and limitations of the NEX-GDDP-CMIP6 ensemble in representing historical temperature, precipitation, and meteorological drought conditions across Iran; (2) assess the robustness, spatial variability, and physical mechanisms of projected temperature and precipitation changes; (3) project future drought probability, intensity, and duration using SPI and SPEI at 1-, 6-, and 12-month timescales; (4) quantify changes in the agreement between precipitation-based and PET-sensitive drought classifications; and (5) determine the relative contributions of precipitation and PET to projected SPEI changes. By connecting model evaluation with drought projections and climate-driver attribution, this study provides a more reliable, process-based basis for drought monitoring, climate risk assessment, and water resource and agricultural adaptation planning in Iran.

## Materials and methods

### Study area

**Fig. 1:**
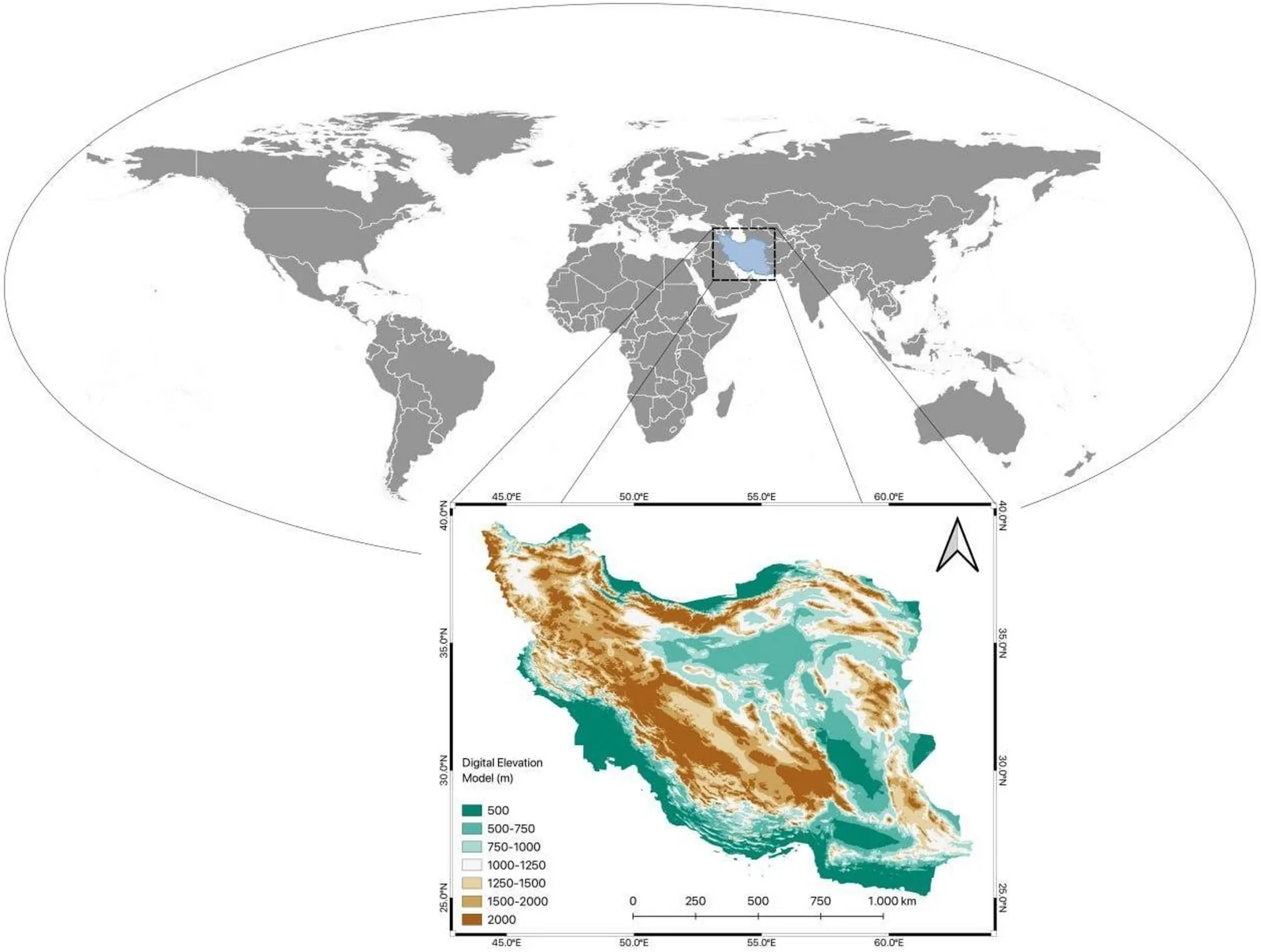
Geographical location of Iran.

Iran spans latitudes 25°–40° N and longitudes 44°–66° E, covering 1,648,195 km^2^ (Fig. 1). Classified largely as arid and semi-arid in the Köppen climate classification^34^, about 88% of its territory comprises desert or dry plains. Precipitation drops markedly from the north (over 1000 mm in the Caspian Sea region) to the central and eastern basins, owing to orographic barriers such as the Alborz and Zagros Mountains^10,35^. Annual mean temperatures range from roughly 9 °C in the north/northwest to about 25 °C –27 °C in the south/southeast, while average precipitation is below one-third of the global mean^36^.

### Dataset

General circulation models (GCMs) are the most common and practical tools for evaluating the cause and mechanism of climate change^37^. GCMs are crucial tools for projecting future climate change impacts on regional and global scales^38^. These models illustrate the most reliable approach for projecting global climate response under different scenarios^39–41^.

This research used global daily high-resolution and bias-corrected GCM datasets from the NEX-GDDP-CMIP6^42^ dataset (accessed December 2023). In the NEX-GDDP dataset, GCMs have been run under several climate scenarios (i.e., SSP1–2.6, SSP2–4.5, SSP3–7.0, and SSP5–8.5). These scenarios represent different future pathways of societal development and associated greenhouse gas emissions. SSP1–2.6 describes a sustainable pathway with low challenges to mitigation and adaptation, leading to low radiative forcing; SSP2–4.5 represents an intermediate scenario with moderate emissions and challenges; SSP3–7.0 outlines a fragmented world with high challenges and higher emissions; and SSP5–8.5 depicts a fossil-fuel-intensive future with very high emissions and radiative forcing^41^.

The developed model for investigating the future of droughts in Iran is based on a multi-model mean of 25 models (see Table S1). The selection of these models was based on the availability of consistent temperature and precipitation data for the selected SSP scenarios (SSP1–2.6, SSP2–4.5, SSP3–7.0, and SSP5–8.5), which are essential for projecting future meteorological droughts in Iran. The added value of NEX-GDDP datasets over native GCMs by incorporating advanced bias correction and spatial downscaling to a high resolution (0.25° × 0.25°) has been demonstrated in many studies^43–46^. For example, NEX-GDDP-CMIP6 multi-model ensemble reproduced a coherent spatial precipitation pattern better than the native CMIP6 GCMs in simulating precipitation in Africa^47^. In addition, the NEX-GDDP datasets have been used successfully in the projection of drought events in the future for different parts of the world^48–52^. To compare and evaluate the capability of the employed multi-model ensemble means, an observation dataset of monthly temperature and precipitation for 51 synoptic stations (see Table S2) in Iran (1985–2014) has been used (www.data.irimo.ir). To align the NEX-GDDP model outputs with observational data, each of the 51 meteorological stations was paired with its corresponding grid cell via bilinear interpolation. This method is commonly used in regional climate model evaluation to ensure spatial consistency between modeled and observed data while minimizing interpolation uncertainty^53–55^.

In assessing the main climate variables and drought occurrences, two 30-year intervals have been used for the historical (1985–2014) and the future (2071–2100) periods. We selected 1985–2014 as the reference period because it is the latest 30-year period covered by the historical GCM simulations, and it captures recent climate variability, including the influence of anthropogenic warming.

### Drought analysis indices

Future drought conditions in Iran were investigated using two widely used indices: the Standardized Precipitation Index (SPI)^56^ and the Standardized Precipitation-Evapotranspiration Index (SPEI)^57^. The SPI relies only on precipitation data, making it ideal for monitoring meteorological droughts^58^. SPEI enhances the capability for evaluating the impact of climate change on droughts by incorporating PET. SPEI is based on the climatic water balance, calculated as precipitation minus PET (P − PET), and integrates temperature effects through PET calculations, which provides a more comprehensive view of how climate change influences drought conditions^57^. The capability of SPI/SPEI in the analysis of future drought conditions has been proven in previous studies^59–61^. Several empirical and physically based methods have been developed to estimate PET, including the Hargreaves–Samani^62^, Thornthwaite^63^, and Penman–Monteith (PM)^64^. Although the PM method is recognized as the recommended method, its application is often limited by the requirement of extensive data, such as wind speed, solar radiation, and humidity. Consequently, the Hargreaves method serves as a robust and suitable alternative to the PM method. Therefore, in this study, PET was estimated using the Hargreaves–Samani method, which employs maximum and minimum air temperatures together with extraterrestrial radiation. Previous studies confirmed that the Hargreaves method is widely recognized as reliable and consistent for arid and semi-arid regions, particularly those in Iran and the Middle East^65–67^.

We applied the climate_indices Python package^68^ to derive SPI and SPEI, a method previously proven effective in multiple drought investigations^69–71^. In this research, the SPI and SPEI indices were fitted to the Pearson Type III probability distribution, as it is widely considered a suitable method adopted in drought studies^72,73^. The choice of the Pearson Type III model enhances the reliability of our drought characterization across Iran’s diverse climatic zones, providing a robust basis for projected data and deriving the final SPI and SPEI values. More details of the SPI, SPEI, and PET calculations are provided in the Supplementary material.

SPI and SPEI values were calculated using the model developed for 1-, 6-, and 12-month timescales. Using SPI/SPEI at 1-, 6-, and 12-month scales captures the dynamics of meteorological drought across different temporal resolutions. The 1-month scale highlights immediate anomalies in precipitation and temperature, the 6-month scale captures seasonal variations, and the 12-month scale reflects broader annual trends, offering a robust framework for analyzing meteorological droughts.

The severity of droughts, as indicated by SPI and SPEI, was classified using the thresholds presented in Table 1, proposed by McKee et al.^56^ and Vicente-Serrano et al.^57^. Moreover, drought characteristics (frequency and duration) were estimated using the equations presented in Table S3.

**Table 1:** Drought severity categories and corresponding thresholds for SPI and SPEI.

Drought severity category	SPI/SPEI threshold
Moderately dry	− 1.5 < SPI/SPEI ≤ − 1.0
Severely dry	− 2.0 < SPI/SPEI ≤ − 1.5
Extremely dry	SPI/SPEI ≤ − 2.0

The probability of drought occurrence was evaluated for historical and projected periods by calculating the ratio of drought months to the total number of months in each timeframe. The duration of a drought event was defined as the continuous sequence of months between its onset and termination. Relative changes were then estimated by comparing future values with those from the historical baseline period. Drought duration values represent the length of individual continuous drought episodes rather than the cumulative number of drought months across separate events. In this study, the distribution parameters of SPI and SPEI were fitted using the historical reference period (1985–2014). These fixed reference-period parameters were then applied to the continuous monthly climate time series for the historical and future periods (1985–2100) to calculate SPI and SPEI. This approach prevents the future climate signal from being incorporated into the standardization process and allows projected changes in drought to be assessed relative to the historical climate baseline. After SPI and SPEI were calculated, drought events were identified as consecutive months during which the index remained below the drought threshold, ensuring that events were not artificially interrupted at the boundary between historical and projected periods. If a drought event was still ongoing in December 2100, it was treated as a right-censored event and excluded from the drought-duration statistics because its complete duration could not be determined from the available record.

To evaluate the capability of the adopted drought indices in representing historical drought conditions over Iran, SPI and SPEI were calculated from observed meteorological station records for the reference period (1985–2014) at 1-, 6-, and 12-month accumulation timescales. The resulting time series were examined to verify whether the indices reproduced the temporal evolution and persistence of historically documented drought events before their application to future drought projections.

### Performance evaluation

The multi-model ensemble’s historical performance (1985–2014) over the study area was evaluated using probability density functions (PDFs) and histograms of 30-year trends for precipitation and temperature. This distribution-based evaluation focused on four key characteristics: (1) central tendency, including agreement in modal and median values; (2) spread and variability, including agreement in distribution width and variance; (3) skewness and overall distributional shape, which is particularly important for precipitation; and (4) tail behavior, reflecting the representation of warm/cold extremes and heavy-precipitation events. This evaluation framework was selected because historical CMIP6 simulations are externally forced but freely evolving coupled climate simulations. Although they are driven by prescribed historical forcings, they are not initialized or constrained to reproduce the observed phasing of internally generated climate variability^74–76^. Therefore, these models’ performance should not be expected to reproduce the exact chronological timing of observed historical meteorological events. This is particularly important for regional hydroclimatic variables, where internal variability can produce substantial differences between simulated and observed trends, extremes, and wet or dry periods, even over multi-decadal timescales^77,78^. As a result, metrics that require direct time-by-time pairing between simulations and observations, including temporal correlation, can provide a misleading assessment of model skill when applied as the primary validation criterion for uninitialized historical climate simulations. Such metrics may penalize expected phase differences in internal variability rather than evaluating the model’s ability to reproduce the statistical characteristics of the climate system^76,78^. Accordingly, the historical evaluation in this study emphasizes whether the ensemble reproduces the broader statistical characteristics of the observed climate rather than the exact timing of individual historical events.

This approach is particularly relevant for drought assessment because SPI and SPEI are standardized indices derived from fitted probability distributions. For SPI, precipitation totals are fitted to a probability distribution and then transformed into standardized values; therefore, the choice and adequacy of the fitted distribution directly affect drought classification^79^. Similarly, SPEI depends on the distributional behavior of climatic water balance. Thus, the ability of the model ensemble to reproduce precipitation and temperature distributions, including their spread, skewness, and tails, is directly related to the reliability of subsequent drought-index calculations. This approach is also consistent with climate model evaluation studies that emphasize that performance metrics should be selected according to the intended application rather than relying on a single universal metric^80^. Furthermore, probability-distribution comparisons provide a useful framework for climate-model assessment because they evaluate broader distributional characteristics rather than only paired values at individual time steps^81^. Consequently, PDFs and histograms were used as the primary evaluation tools to assess whether the NEX-GDDP-CMIP6 ensemble captures the distributional properties and variability of the observed baseline climate.

The statistical analysis to quantify differences between SPI and SPEI results was conducted using Cohen’s Kappa (κ) statistic^82^ as a measure of similarity between their categorical drought classifications. κ values range from − 1 (complete divergence) to 1 (perfect correspondence), with higher values indicating closer correspondence between indices. The comparison was performed for each CMIP6 scenario (SSP1–2.6, SSP2–4.5, SSP3–7.0, and SSP5–8.5) and at 1-, 6-, and 12-month timescales using ensemble-mean drought classifications across Iran. This approach allowed a quantitative assessment of how the inclusion of PET alters the relationship between SPI and SPEI under future climate conditions.

To evaluate the magnitude and robustness of projected hydroclimatic changes, we applied two complementary statistical approaches. First, spatial changes were calculated for each GCM, SSP scenario, and grid cell. Temperature change was expressed as absolute change (ΔT, °C), while precipitation change was expressed as relative change (ΔP, %) with respect to the historical baseline. The ensemble-mean changes were mapped, and hatching was used to identify grid cells where the projected change satisfied the statistical significance and ensemble-robustness criteria. It should be noted that the multi-model ensemble was not treated as a formal random sample from a well-defined population of all possible climate futures. Therefore, statistical tests were used to evaluate changes within individual model simulations and grid cells, while ensemble robustness was assessed descriptively based on the consistency, sign agreement, and spatial extent of significant changes across the available GCMs. This approach avoids over-interpreting ensemble-level p-values and instead emphasizes whether projected signals are consistent across models^83^. Additionally, projected temporal trends were evaluated for each GCM and SSP scenario. The Mann–Kendall test^84^ was applied to detect increasing or decreasing trends without assuming normality, making it suitable for hydroclimatic variables with high interannual variability. Sen’s slope estimator^85^ was used to quantify trend magnitude because it is less sensitive to outliers than ordinary least squares regression. Temperature trends were reported in °C decade⁻1, and precipitation trends were reported in % decade⁻1. Trends were considered statistically significant at p < 0.05. Together, these analyses allowed us to evaluate both the spatial robustness of projected changes and the consistency of temporal trends across individual GCMs.

## Results

### Historical performance of the NEX-GDDP-CMIP6 ensemble

**Fig. 2:**
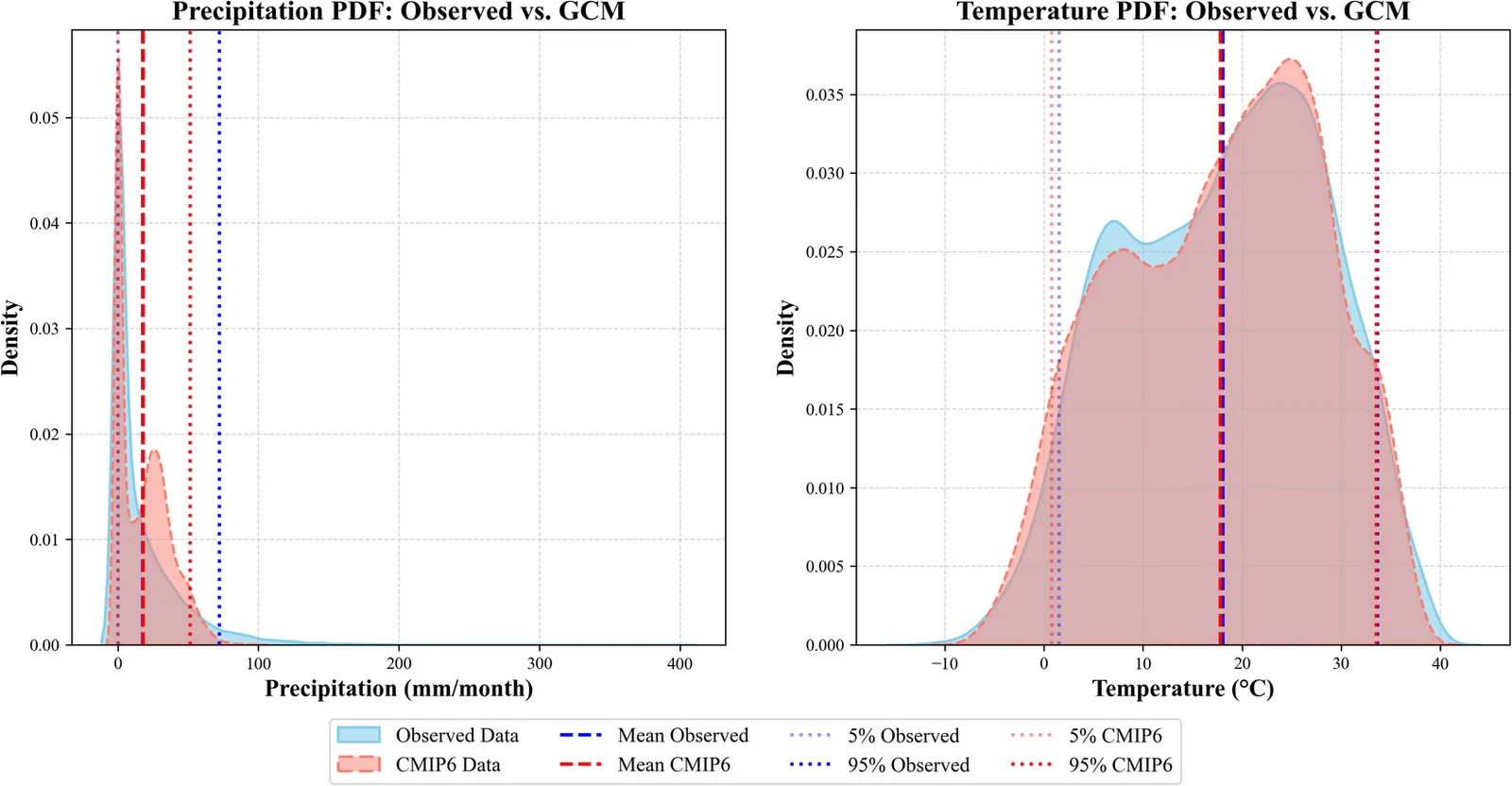
Comparison of probability density functions for observed and CMIP6-modeled precipitation (a) and temperature (b).

**Fig. 3:**
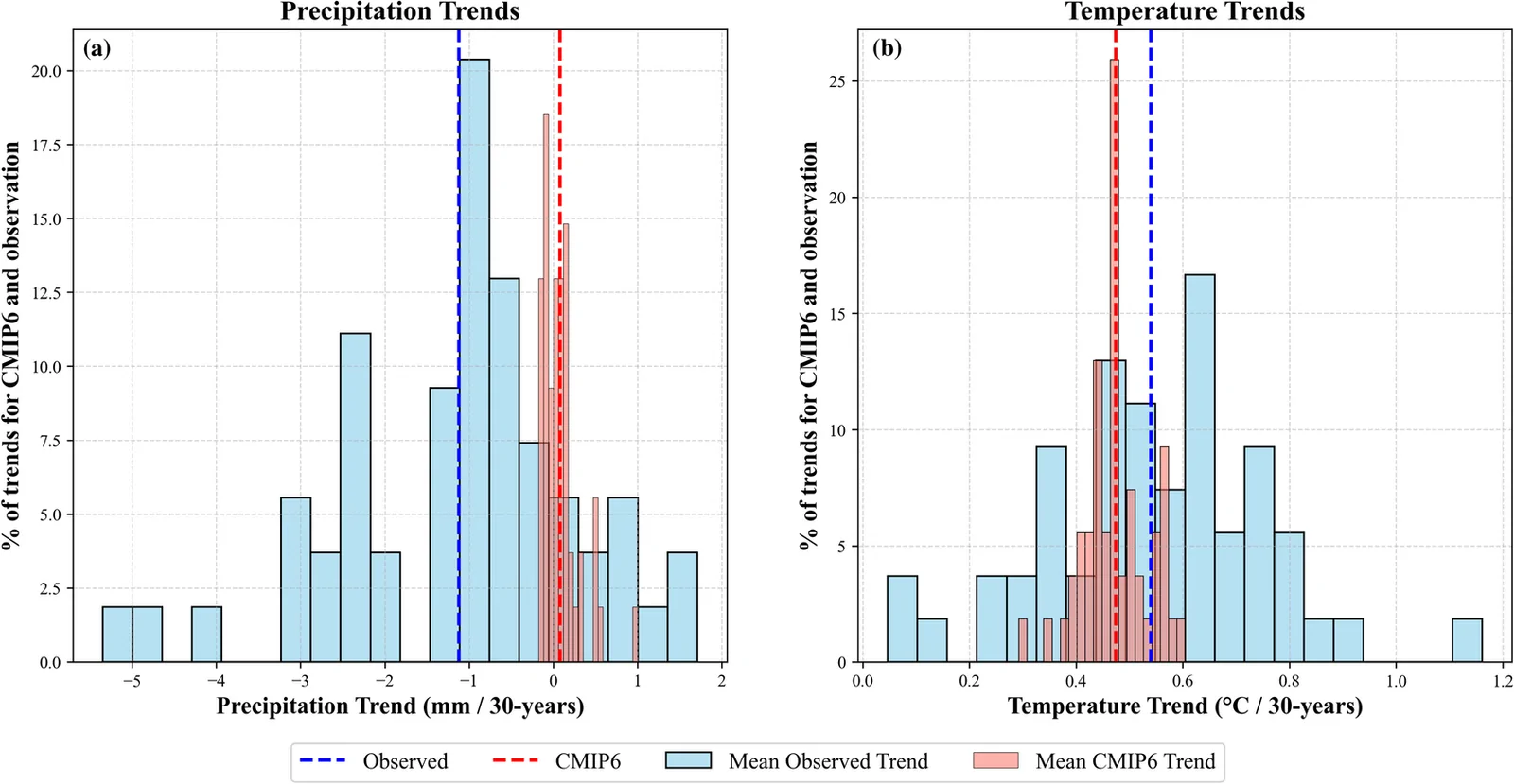
Histogram of precipitation (a) and temperature (b) trends over a 30-year (1985–2014) period for observed and multi-model CMIP6 simulations.

The multi-model ensemble’s performance in simulating precipitation based on PDFs (Fig. 2a) showed close agreement in the mean performance of model data. However, extreme precipitation estimates differ slightly from observations. While the model captures the general trend of precipitation distribution, it underestimates the intensity of extreme precipitation events. It is important to underscore that the inherent uncertainty of precipitation data due to its complex nature is still challenging for researchers in climate change studies^86,87^. By comparing the PDFs of the multi-model mean and observation data (Fig. 2b), the results show relatively good performance in simulating temperature distributions compared to the observations. The temperature PDFs indicate that the model captures the general shape and range of observed temperatures. For instance, the 5th percentile temperature has a negligible difference between observed and model data. The histogram for the distribution of 30-year trends (1985–2014) for precipitation and temperature in observations and CMIP6 model simulations is depicted in Fig. 3. For precipitation (Fig. 3a), the observed trends are more negative than the mean CMIP6 trend, indicating a stronger drying trend in observations compared to the model ensemble. This suggests that CMIP6 models may underestimate the observed decline in precipitation. The trends in temperature (Fig. 3b) from the observation data are higher than the mean CMIP6, indicating that the observed warming is stronger than that predicted by CMIP6 models. CMIP6 model trends are more concentrated around lower warming values, whereas observed trends show a broader range with a tendency toward higher warming. These results indicate that CMIP6 models, which exhibit a slight underestimation, capture the general warming trend.

To assess the potential differences between observations and CMIP6 data in Iran’s complex terrain, an additional evaluation was performed by grouping the 51 synoptic stations into lowland (0–500 m), mid-altitude (500–1000 m), high-elevation (1000–2000 m), and high mountain (> 2000 m) classes (Fig. S1). The cumulative distribution functions show that temperature is generally well represented across elevation classes, with relatively small differences between observed and modeled distributions. In contrast, precipitation shows distributional differences, particularly in the mid-altitude, high-elevation, and high mountain classes. This result is expected because precipitation in mountainous regions is strongly affected by orographic lifting, rain-shadow effects, exposure, and steep local elevation gradients that may not be fully resolved within a 0.25° grid cell. Nevertheless, the elevation-based comparison indicates that the NEX-GDDP-CMIP6 ensemble captures the broad distributional behavior of temperature and precipitation across Iran’s elevation zones, supporting its use for national-scale and regional drought assessment**.**

In addition to evaluating the historical performance of the climate variables, the capability of the adopted drought indices was assessed using observed station records (Fig. S2). Both SPI and SPEI reproduce the temporal variability of historical drought conditions across Iran, with negative anomalies evident during the late 1990s to early 2000s. This period corresponds to the well-documented 1998/1999–2002 drought episode, which previous Iran-focused studies identified as one of the most persistent and spatially extensive droughts in recent decades^11,88,89^. The signal is more clearly expressed at the 6- and 12-month timescales, indicating that the adopted indices capture the persistence of multi-month drought conditions. These results provide additional confidence that SPI and SPEI realistically represent observed drought variability and are suitable for projecting future drought characteristics in Iran.

### Projection of future temperature and precipitation

**Fig. 4:**
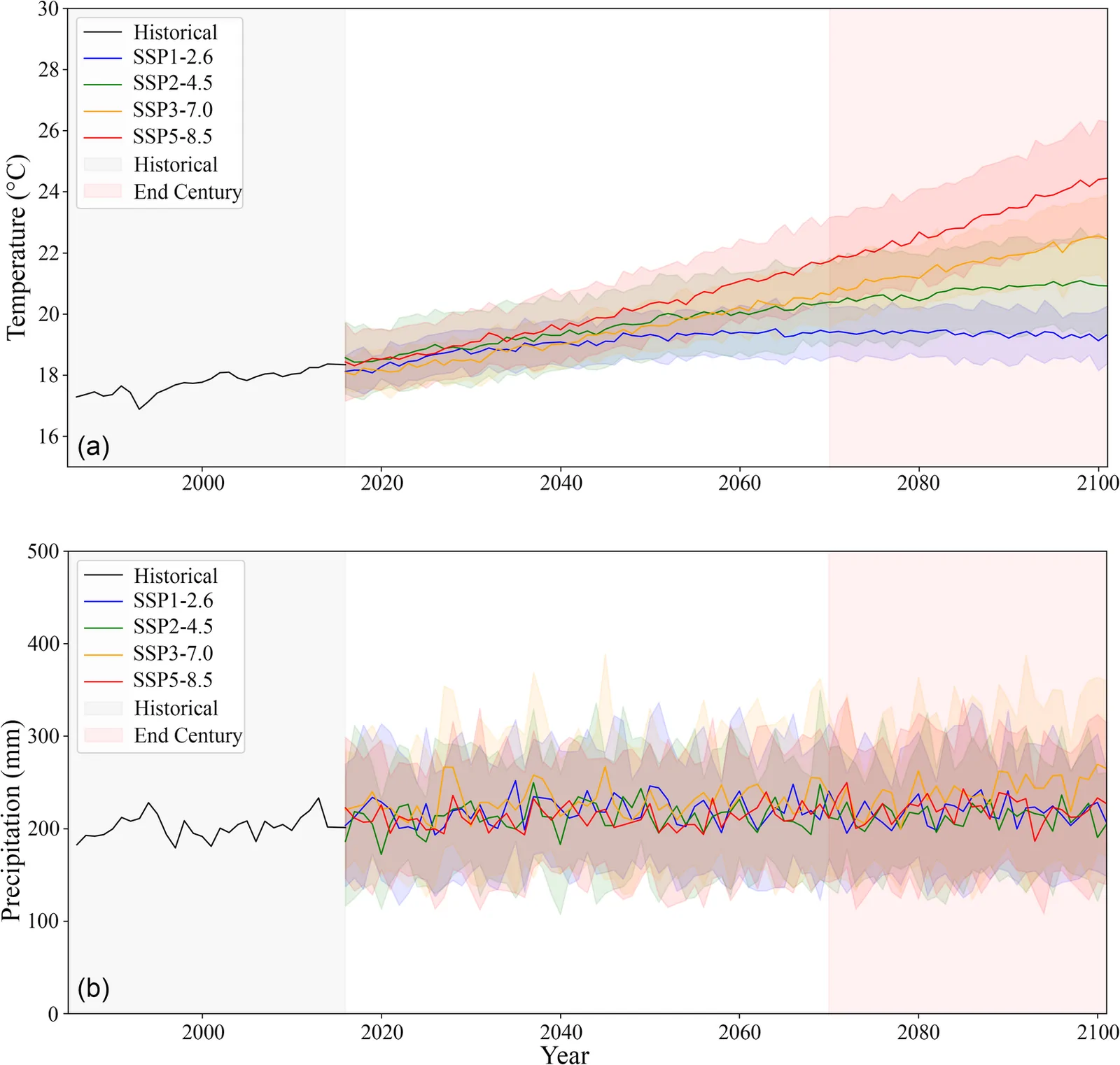
Time-series of historical and projected changes in (a) average annual temperature and (b) annual total precipitation in Iran in 1985–2100 (*Key: For the historical period, only the ensemble mean of 25 GCMs is shown, whereas for the SSP scenarios the shaded bands represent the inter-model uncertainty ranges).

Based on the created model, projections of temperature and precipitation were done to evaluate the occurrence of droughts in Iran under SSP scenarios. The time series of temperature and precipitation variations in Iran for historical and projected periods under SSP scenarios is depicted in Fig. 4.

The results for year-to-year temperature changes (Fig. 4a) indicate that for the future period (2015–2100), temperatures are expected to increase under all SSP scenarios. The mean annual temperature in Iran was 17.75 °C during the historical period (1985–2014) and is projected to increase to 19.3, 20.9, 22.4, and 24.4 °C during 2071–2100 under SSP1–2.6, SSP2–4.5, SSP3–7.0, and SSP5–8.5, respectively. Analysis of changes in annual precipitation (Fig. 4b) reveals that during the historical period (1985–2014), precipitation fluctuated around an average of approximately 200 mm. For the future period (2015–2100), under all scenarios (SSP1–2.6, SSP2–4.5, SSP3–7.0, and SSP5–8.5), precipitation trends remained the same as in historical periods, ranging from 150 to 300 mm with an average of 200 mm. However, precipitation slightly increased at the end of the twenty-first century (2071–2100), particularly for the SSP3–7.0 scenario.

Evaluating uncertainty across 25 GCMs (Fig. 4) reveals that different models underestimate or overestimate future projections of temperature and precipitation. For example, considering the SSP1–2.6 scenario at the end of the twenty-first century, the multi-model mean for annual temperature across the 25 models is 19.3 °C, whereas the TaiESM1 model projects a substantially higher value of 21.9 °C. At the same time, assessment of the SSP5–8.5 scenario reveals pronounced variations in precipitation projections. While the multi-model mean for annual precipitation is 220 mm, individual models such as ACCESS-CM2 (402 mm) and MRI-ESM2-0 (114 mm) show a wide spread of projections within the ensemble.

The spatial robustness analysis shows widespread warming across Iran under all SSP scenarios (Fig. S3). Temperature changes are positive throughout the country, and nearly all grid cells are hatched, indicating that the warming signal is spatially extensive and robust across the GCM ensemble. Precipitation changes show a more heterogeneous response than temperature (Fig. S3). Although the ensemble-mean signal indicates increased precipitation in several regions and scenarios, the spatial extent of hatched areas varies among SSP scenarios. The area-mean trend analysis further supports this contrast between temperature and precipitation (Fig. S4). Temperature trends are positive across all GCMs and SSPs, with most models showing statistically significant Mann–Kendall trends. In contrast, precipitation trends show larger inter-model spread, with both positive and negative trends occurring within the same SSP.

To further evaluate the seasonal structure of inter-model spread, Fig. S5 presents the monthly climatology of temperature and precipitation for the ensemble mean and the 25 individual NEX-GDDP-CMIP6 models during the historical period. The models show consistent seasonal temperature behavior throughout the year. For precipitation, the spread is larger during the wet season from late autumn to spring, while model agreement is stronger during the dry summer months when precipitation is minimal across most of Iran. This pattern highlights the greater natural variability of precipitation than of temperature and supports interpreting precipitation-based drought results alongside the ensemble spread.

**Fig. 5:**
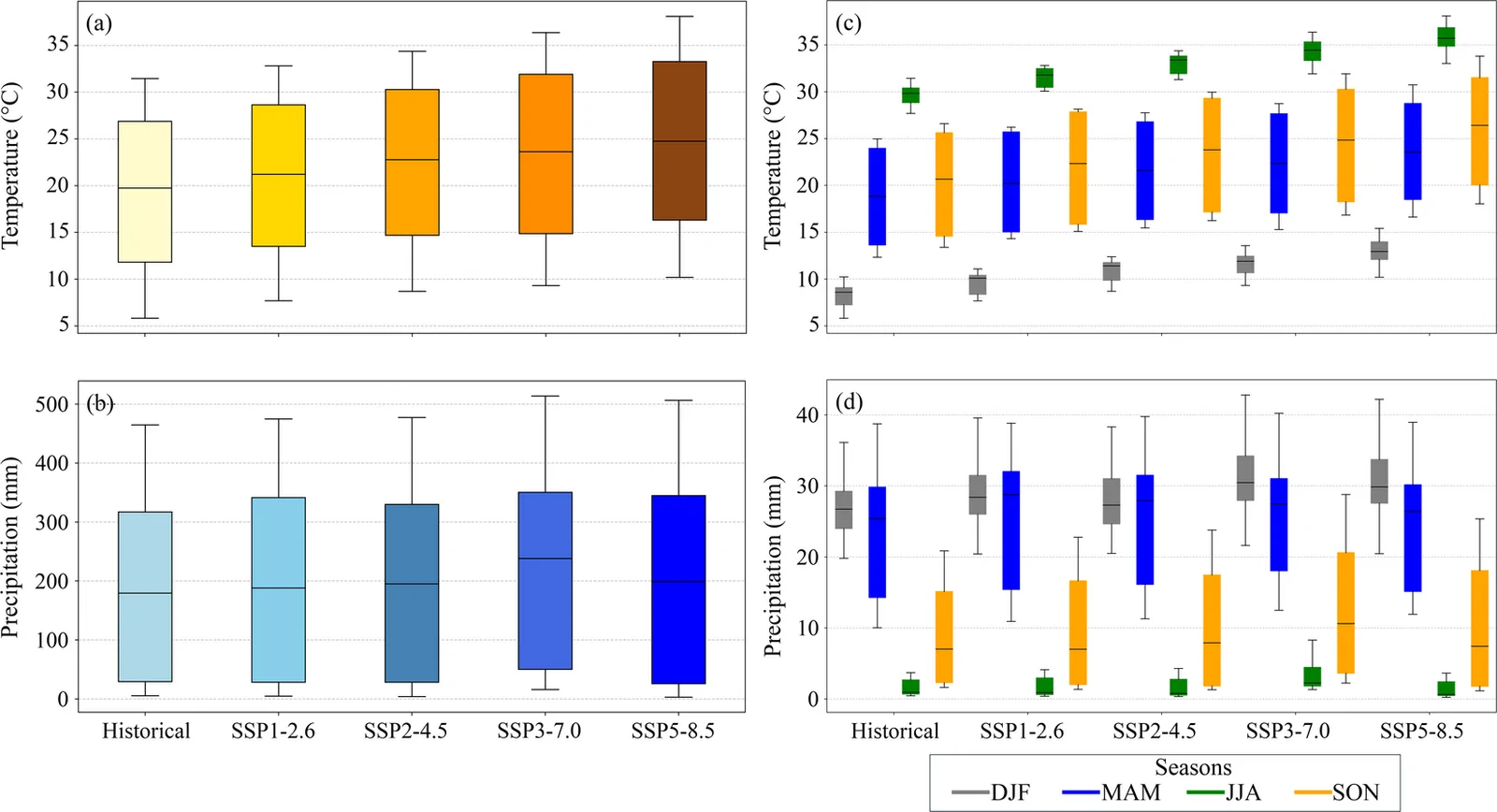
Changes in the (a) mean annual temperature, (b) annual precipitation, (c) seasonal temperature, and (d) seasonal precipitation in Iran, covering both historical (1985–2014) and projection (2071–2100) periods.

The boxplot of annual and seasonal changes in temperature and precipitation is illustrated in Fig. 5. The estimated annual change in temperature (Fig. 5a) for the projected periods shows a clear and consistent increase under the SSP scenarios relative to the 1985–2014 period. The median temperature under the SSP5–8.5 and SSP3–7.0 scenarios is expected to reach ~ 25 °C, compared with ~ 20 °C during the historical period. The largest temperature increase is expected during the summer (JJA) and under the SSP3–7.0 and SSP5–8.5 scenarios, with median temperatures exceeding 30 °C by 4–6 °C relative to the historical period. Annual total precipitation (Fig. 5b) shows variations under SSP scenarios, with only a visible increase under the SSP3–7.0 scenario. Seasonal changes in precipitation (Fig. 5d) show a slight increase in precipitation for different seasons. In the summer (JJA), the precipitation under SSP scenarios is almost the same as in the historical period, with only an increase under the SSP3–7.0 scenario. In the autumn (SON) and winter (DJF), precipitation is expected to increase slightly under all scenarios compared to the historical period.

**Fig. 6:**
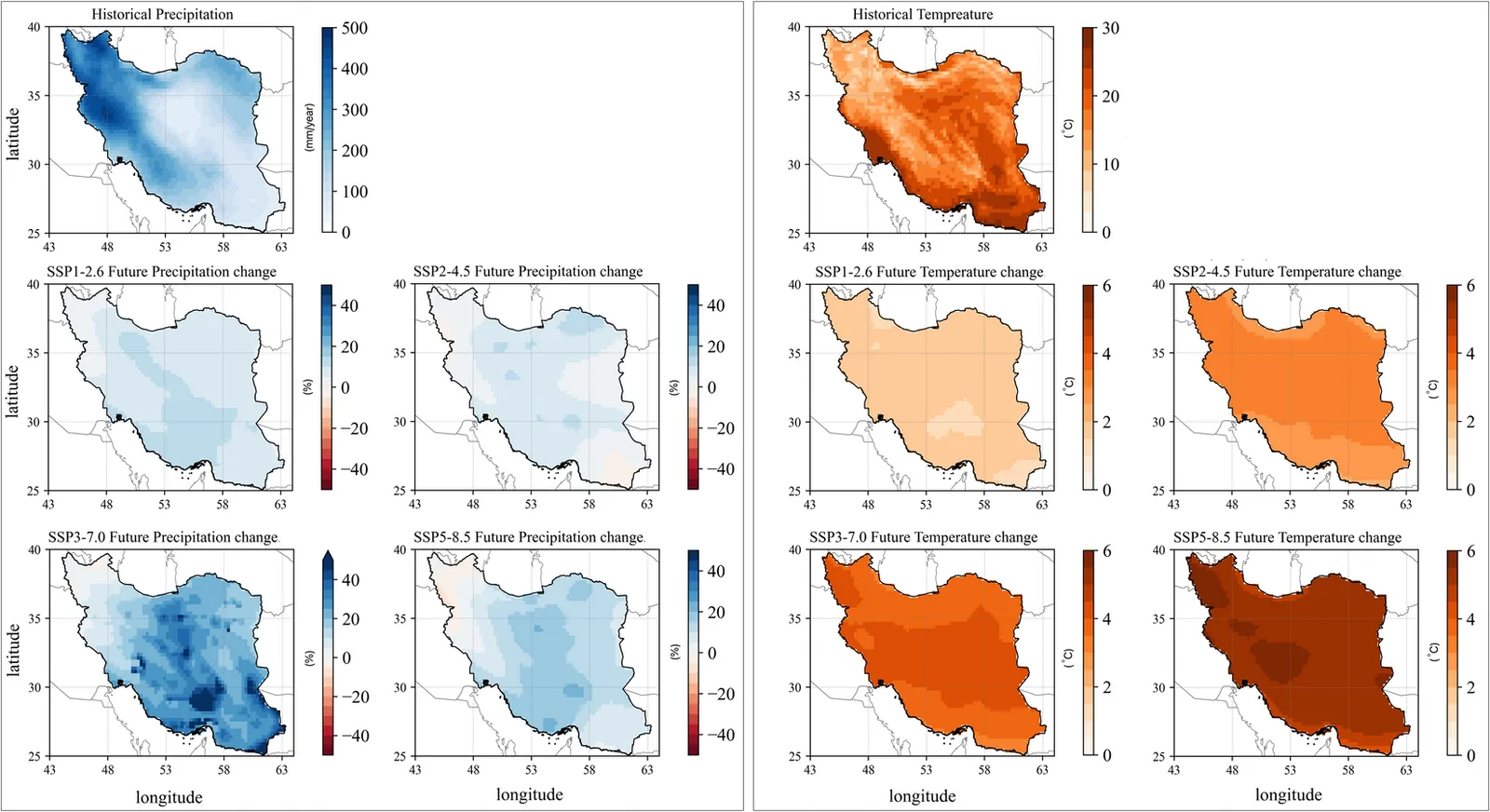
Spatial distribution of mean annual precipitation (left) and temperature (right) over Iran for the historical baseline (1985–2014) and projected changes for the future period (2071–2100) under SSP scenarios (SSP1–2.6, SSP2–4.5, SSP3–7.0, and SSP5–8.5).

The spatial distributions of temperature and precipitation over Iran are presented (Fig. 6). During the historical baseline period (1985–2014), temperatures across Iran ranged from approximately 5 °C to 30 °C, with the lowest values in the northern highlands, particularly within the Alborz and Zagros mountain ranges, and the highest in the southern and southeastern regions. In the future period (2071–2100), under SSP scenarios, relatively homogeneous increases in temperature are projected for Iran. In the SSP1–2.6 scenario, temperatures are projected to increase slightly, by 1–2 °C, particularly in the northern regions. The SSP2–4.5 scenario indicates a warming of 2–4 °C, particularly in central areas. The most extreme temperature increases will occur under the SSP3–7.0 and SSP5–8.5 scenarios, with temperature changes of 4–6 °C in most parts of the country.

The spatial distribution of precipitation during the historical period (1985–2014) demonstrates that the highest precipitation (∼500 mm) is concentrated in the northern regions, particularly near the Caspian Sea, reflecting the influence of humid climatic conditions in this area^90^. In contrast, the central and southeastern regions receive significantly lower precipitation due to the arid and semi-arid conditions, with values below 100 mm. The central plateau and southern desert regions exhibit the lowest precipitation range, with some areas experiencing zero precipitation. Projected changes in annual precipitation over Iran for the future period (2071–2100) relative to the historical period (1985–2014) show that under the SSP1–2.6 and SSP2–4.5 scenarios, precipitation is expected to increase (up to + 15%). The SSP3–7.0 scenario shows more noticeable variability, with average increases between + 20 and 40% in central, southern, and eastern regions. In contrast, under the SSP5–8.5 scenario, precipitation will decrease (up to − 7%) in the northwest regions.

### Projected changes in drought

**Fig. 7:**
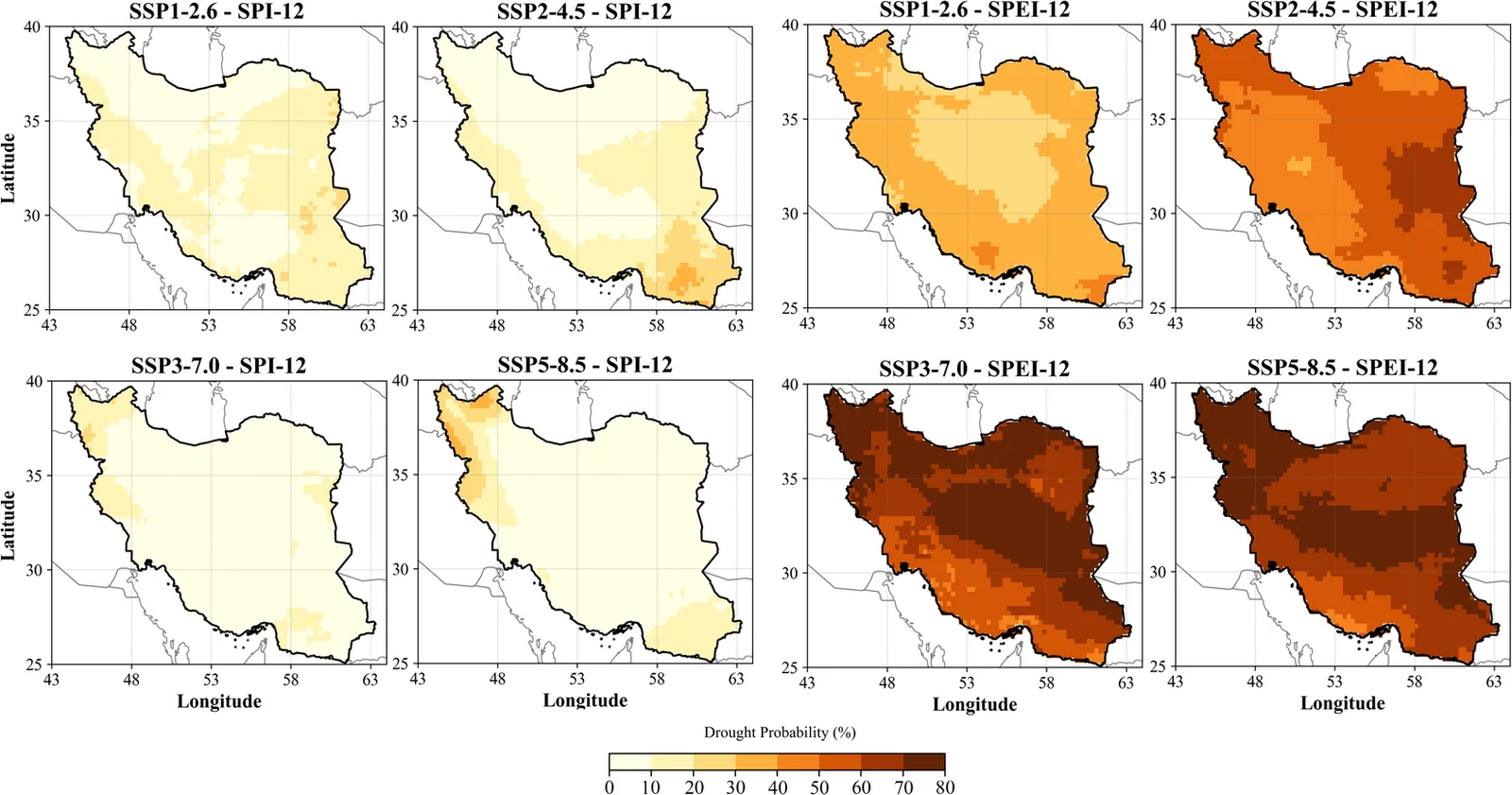
Spatial distribution of drought probability in Iran estimated by SPI-12 (top) and SPEI-12 (bottom) for the future period (2071–2100) under SSP scenarios.

Drought conditions for 2071–2100 were assessed using SPI and SPEI at 1-, 6-, and 12-month accumulation timescales (Figs. S6–S10 and Fig. 7). Results demonstrated that under the SSP1–2.6 scenario for SPI-1 (Fig. S6), the drought probability is in the range of 20–40%, mostly in southeastern and southern regions, while the rest of the country remains below 20%. In the mid-term (SPI-6), drought probability is expected to increase 10–20%, with central and southeastern regions becoming more affected than northern parts (Fig. S7). Long-term droughts (SPI-12) show drought probabilities of 20–30% (Fig. 7). Under SSP2–4.5, drought probability for SPI follows relatively the same trends as the SSP1–2.6 scenario, with slight differences. Under SSP3–7.0 and SSP5–8.5 scenarios, droughts are more frequent in the northwest and southeastern parts of the country. Under the SSP3–7.0 scenario, droughts for SPI-12 in the northwest are projected to reach 20%–40% while exceeding 40% under the SSP5–8.5 scenario for SPI-6 (Fig. S7) and SPI-12 (Fig. 7).

Under SSP1–2.6, drought probability in SPEI-1 (Fig. S6) is relatively low, ranging from 10 to 30%, while it is expected to increase slightly to 20%–40% for SPEI-6 (Fig. S7) and 30%–50% for SPEI-12 (Fig. 7). For SPEI droughts, SSP2–4.5 scenario shows an intensification of drought probability, particularly in central and southeastern Iran, where SPEI-1 (Fig. S6) frequencies range between 30 and 45%, and SPEI-6 (Fig. S7) and SPEI-12 (Fig. 7) increase up to 65%, demonstrating a moderate increase in drought persistence. Drought probability becomes severe and widespread under the SSP3–7.0 and SSP5–8.5 scenarios. For SPEI-1 (Fig. S6), most regions of the country experience frequencies between 45 and 70%, with the southern and central regions frequently exceeding 65%. For SPEI-6 (Fig. S7) and SPEI-12 (Fig. 7), drought probabilities even exceed 65% across most regions, with probabilities exceeding 80% in southern and central Iran, indicating persistent drought conditions.

The spatial distribution of average drought duration under SSP scenarios for SPI/SPEI (Figs. S8–S10) shows that for almost all scenarios (SSP1–2.6, SSP2–4.5, SSP3–7.0, and SSP5–8.5), the drought duration remains relatively low in SPI-1 (Fig. S8) and SPI-6 (Fig. S9), ranging between 2 and 6 months. In SPI-12 (Fig. S10), under the SSP1–2.6 and SSP2–4.5 scenarios, the northwestern, central, and eastern areas experience prolonged droughts, lasting 8–10 months. For SSP3–7.0 and SSP5–8.5 scenarios and SPI-12, results indicate that drought durations in the southern parts exceed 10 months.

The analysis of drought duration for SPEI for SSP scenarios (Figs. S8–S10) reveals that drought durations for SPEI-1 under SSP1–2.6 and SSP2–4.5 scenarios are within a relatively low range (less than 5 months). In addition, for SPEI-6, drought duration under SSP1–2.6 and SSP2–4.5 scenarios is slightly increased to 6–10 months. The increase in drought duration under the SSP1–2.6 and SSP2–4.5 scenarios for SPEI-12 is relatively significant compared to SPEI-1 and SPEI-6, with most regions experiencing durations exceeding 10 months. The most significant increase in drought duration is expected under the SSP3–7.0 and SSP5–8.5 scenarios at SPEI-12 (Fig. S10), where drought durations exceed 14 months across most regions. Under SSP2–4.5, SSP3–7.0, and SSP5–8.5 scenarios, SPEI-12 drought durations increase substantially, with most regions experiencing events lasting at least 10 months and exceeding 14 months across much of the country.

Another interesting finding is related to the spatial distribution of drought durations for extreme droughts (SPI/SPEI <  − 2.0) under SSP scenarios (Fig. S11). For SPI-1, under all SSP scenarios, extreme drought durations are slightly increased, primarily in the central and southern regions, with values generally ranging from 2 to 6 months. For SPI-6, drought duration increases only in the northwest/southwest under the SSP3–7.0 and SSP5–8.5 scenarios. The prolonged duration of extreme droughts for SPI is expected to occur in the northwest and southeast of Iran under the SSP5–8.5 scenario, reaching 10–12 months. For SPEI-1, the duration of extreme droughts across all SSP scenarios ranges from 2 to 5 months. Under the SSP1–2.6 scenario, the duration of extreme droughts is projected to increase in central Iran, particularly under the SPEI-12 index, with events lasting approximately 8–12 months in these areas. Under SSP2–4.5, SSP3–7.0, and SSP5–8.5 scenarios at SPEI-12 scales (Fig. S11), the duration of extreme droughts is higher than in other timescales and scenarios. Results show that the northeastern and southwestern parts of Iran are projected to experience a significant increase in extreme drought events, reaching 12–14 months. Under SSP3–7.0 and SSP5–8.5 scenarios, the SPEI-12 results show a northward expansion of prolonged drought from the arid central plateau into the climatic transition zone along the Alborz Mountains and the southern Caspian margin. Droughts become more persistent in these northern transition areas, while the longest projected durations occur in northeastern Iran, reaching approximately 12–14 consecutive months; thus, the high-emission signal reflects both intensification within the arid interior and an expansion of persistent drought toward the more humid northern rim.

**Fig. 8:**
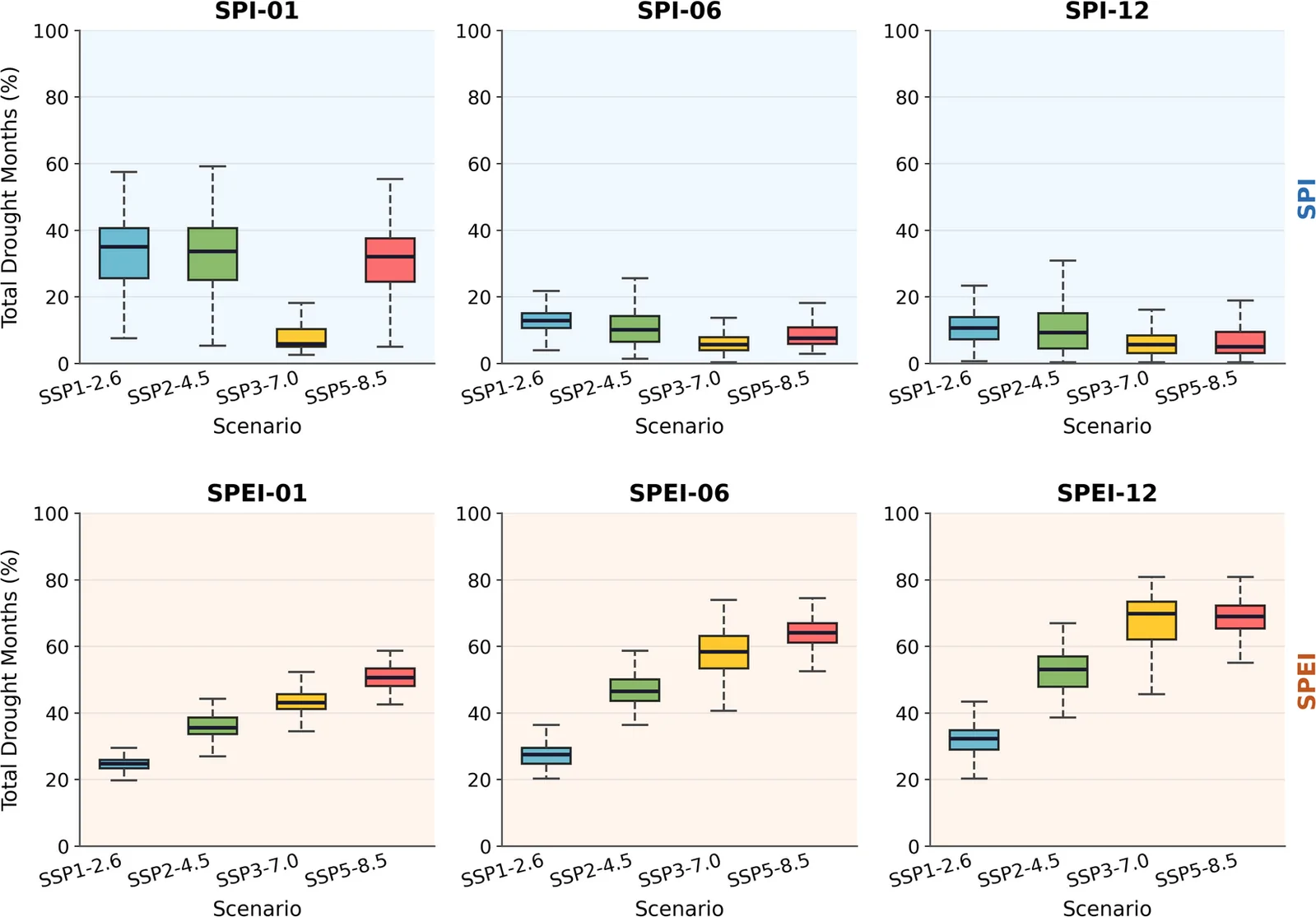
Drought probability variations estimated by SPI (top) and SPEI (bottom) under SSP scenarios in Iran for the projected period (2071–2100).

To summarize the results, the variability of drought duration and probability was compared using boxplots for SPI and SPEI under SSP scenarios. By comparing the probability of droughts for SPI (Fig. 8), the results indicate that in the SPI-1, SSP1–2.6, SSP2–4.5, and SSP5–8.5 scenarios, the drought probability is similar (~ 35%), whereas the SSP3–7.0 scenario has a very low probability (~ 7%). SPI-6 shows a lower drought probability, with SSP1–2.6, SSP2–4.5, and SSP5–8.5 ranging between 14 and 20%, while it is particularly low for the SSP3–7.0 scenario (approximately 3%). For SPI-12, SSP1–2.6 and SSP2–4.5 have a probability of almost 10%, while SSP3–7.0 and SSP5–8.5 experience approximately 3%. In general, for SPI, under the SSP3–7.0 scenario, the probability of droughts is consistently lower, while SSP5–8.5 shows a higher frequency of more prolonged droughts. It appears that, under SSP3–7.0, fewer SPI droughts are associated with increased precipitation in Iran.

The SPEI results show a clear increase in drought probability from SSP1–2.6 to SSP5–8.5 scenarios across all accumulation timescales (Fig. 8). Under short-term conditions (SPEI-1), drought probability remains below 30% under SSP1–2.6, while it gradually increases to nearly 55% under SSP5–8.5, indicating a growing probability of meteorological drought events. The medium-term index (SPEI-6) results show a more pronounced increase, with the median drought probability rising from approximately 30% under SSP1–2.6 to over 60% under SSP5–8.5, indicating a stronger persistence of moisture deficits. For long-term (SPEI-12) drought conditions, which become dominant under high-emission scenarios (SSP3–7.0 and SSP5–8.5), the exceedance probability exceeds 70%. These consistent increases across timescales emphasize a substantial rise in drought occurrences for the end of the century. Overall, SPEI indicates longer and more persistent drought conditions than SPI, particularly under the higher-emission scenarios.

**Fig. 9:**
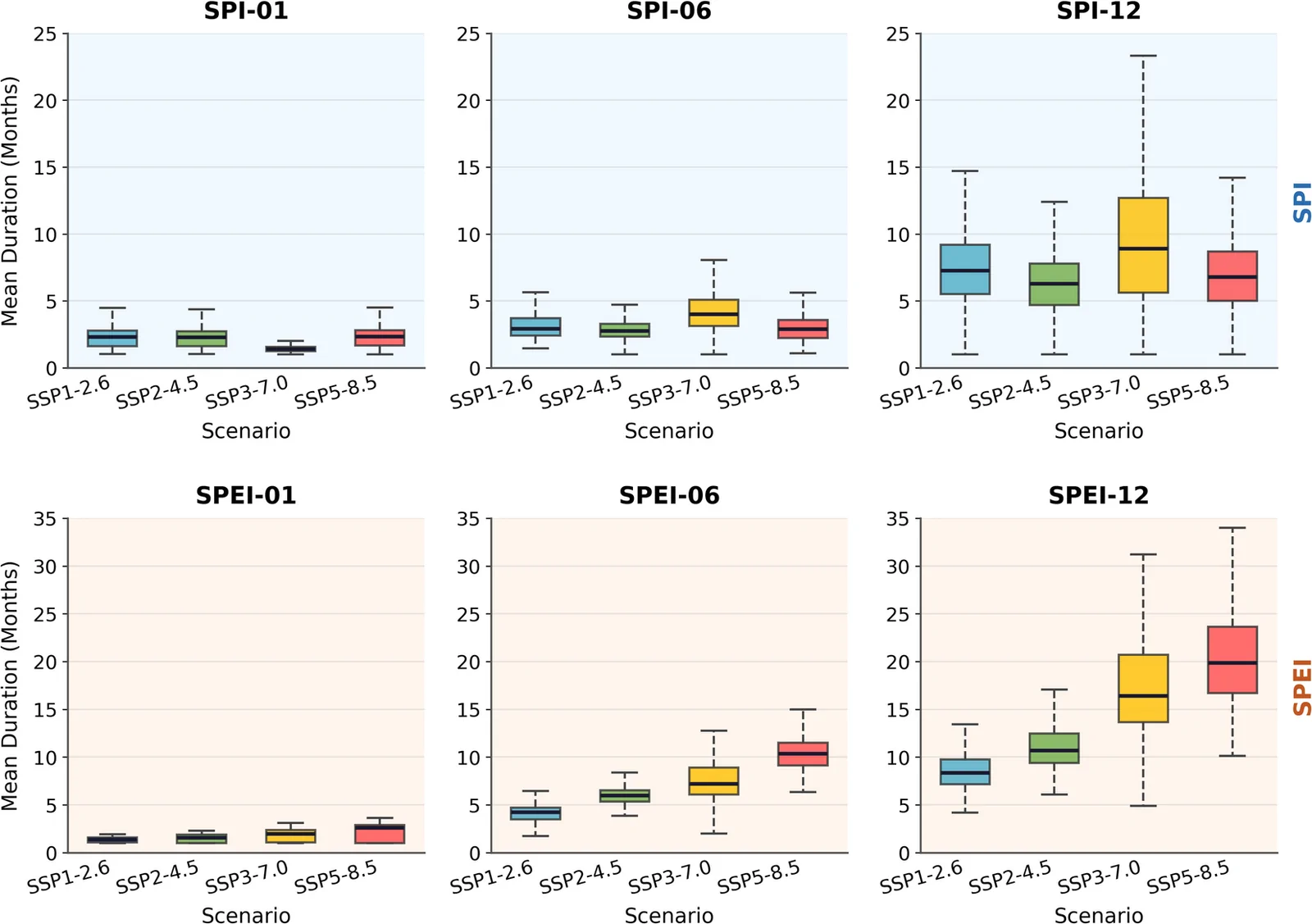
Drought duration variations estimated by SPI (top) and SPEI (bottom) under SSP scenarios in Iran for the projected period (2071–2100).

Comparing results for the duration of droughts under SSP scenarios (Fig. 9) demonstrated that under SSP1–2.6, SSP2–4.5, and SSP5–8.5 scenarios, SPI-1 and SPI-6 have almost similar median durations (~ 3 months), while SSP3–7.0 shows the shortest (~ 2 months) in SPI-1 and highest (~ 8 months in SPI-12). For SPI-12, median drought durations are higher than for SPI-1 and SPI-6 across all scenarios, ranging from approximately 7 to 10 months.

SPEI drought durations indicate that SPEI-1, across all scenarios, has a median of less than 5 months. This range increases slightly for SPEI-6, where drought durations will reach approximately 8 and 10 months in the SSP3–7.0 and SSP5–8.5 scenarios, respectively. The increase in drought durations under the SSP3–7.0 and SSP5–8.5 scenarios in SPEI-12 is substantial, with the median exceeding 20 months in both scenarios.

In addition to the far-future drought probability and duration results, mid-century projections for 2041–2070 are presented and summarized as boxplots of SPI and SPEI under the SSP scenarios (Fig. S12). For SPI, drought probability and duration generally remain relatively low at longer timescales, particularly under SSP3–7.0. In contrast, SPEI shows a clearer increase in both drought probability and mean duration from SSP1–2.6 to SSP5–8.5, especially at the 6- and 12-month timescales. Overall, these results indicate that SPEI projects more frequent and persistent drought conditions than SPI even during 2041–2070.

The seasonal changes in drought probability, as presented in Fig. S13, indicate that, for the SPI, a 1-month drought probability increases during summer (JJA) under the SSP2–4.5 and SSP5–8.5 scenarios, whereas drought probability remains comparatively low and largely uniform across seasons at the 6- and 12-month scales. For the SPEI, drought probability is higher in spring (MAM) and summer (JJA) at SPEI-1 under all scenarios. At the 6- and 12-month scales, drought probability becomes high and similar across seasons, particularly for summer (JJA) and autumn (SON), reflecting more persistent drought conditions. The seasonal SPEI drought patterns are consistent with projected temperature and precipitation changes, with the highest drought probabilities occurring in summer (JJA), which correspond to higher temperatures and generally low precipitation across the SSP scenarios.

**Fig. 10:**
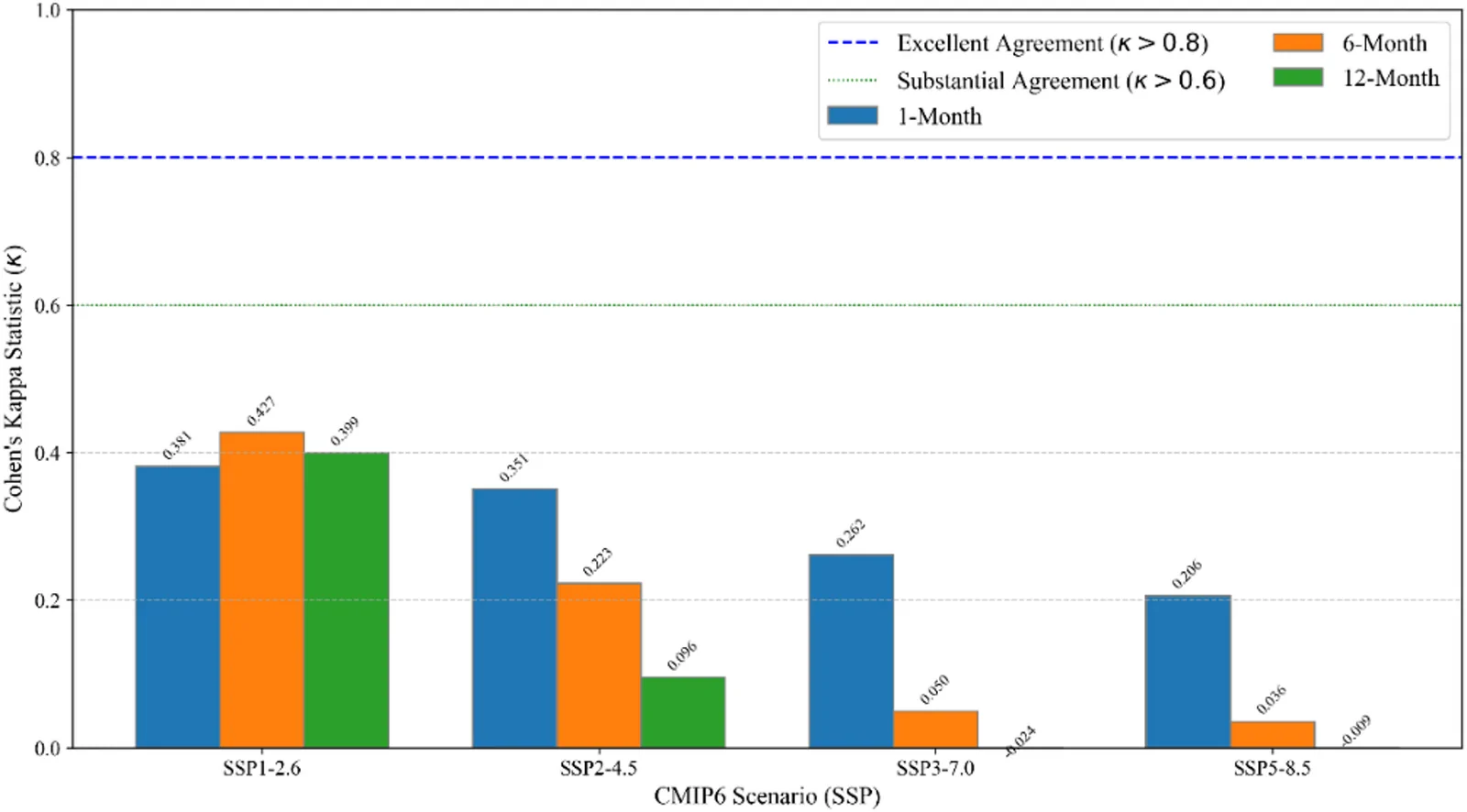
Statistical comparison between SPI and SPEI drought classifications under SSP scenarios and timescales, expressed using Cohen’s Kappa (κ) statistics.

To statistically compare the differences in SPI and SPEI drought projections (Fig. 10), the results were analyzed using Cohen’s Kappa for various SSP scenarios and timescales. Results show that under SSP1–2.6, agreement is moderate (κ ≈ 0.38–0.43), indicating similar drought categorization between the two indices. However, the agreement progressively weakens under higher-emission scenarios, dropping to κ ≈ 0.20–0.26 for SSP5–8.5, particularly at longer timescales. This decline highlights the growing divergence between precipitation-based (SPI) and temperature-sensitive (SPEI) metrics as warming intensifies. The results confirm that temperature-driven increases in PET intensify drought severity in future periods, making SPEI more responsive to climate-warming effects than SPI.

The decline in Cohen’s Kappa agreement between SPI and SPEI under the SSP3–7.0 and SSP5–8.5 scenarios provides an important clarification beyond the magnitude of projected drought change. It indicates that SPI and SPEI drought classifications increasingly diverge as warming intensifies. In practical terms, this means that precipitation deficits alone may no longer fully reflect future drought conditions, because rising temperatures and PET can intensify water stress even when precipitation changes are small or spatially variable. SPI remains useful for identifying precipitation-driven meteorological drought, whereas SPEI provides additional information on the role of warming-driven evaporative demand. Therefore, the declining agreement between the two indices suggests that SPI and SPEI should be used together rather than treated as the same measure in future drought monitoring and early-warning systems in Iran.

### Drivers of changes in drought

To identify the dominant climatic drivers of projected drought variations in Iran, changes in SPEI were decomposed into contributions from precipitation and PET. This attribution framework quantifies the relative contributions of precipitation and PET to projected changes in SPEI. Figures S14–S16 present the distribution of precipitation, PET, and total attribution effects across different timescales (SPEI-1, SPEI-6, and SPEI-12) and scenarios (SSP1–2.6, SSP2–4.5, SSP3–7.0, and SSP5–8.5), while Figures S15 and S16 illustrate their spatial patterns across the country.

The boxplots in Figure S14 demonstrate the consistent dominance of PET-related effects over precipitation-related effects in driving SPEI changes. Across all SSP scenarios and timescales, PET-related reductions in SPEI are notably stronger than those resulting from precipitation changes. This contrast becomes increasingly pronounced under high-emission scenarios and longer accumulation periods, particularly SPEI-6 and SPEI-12. Under the SSP3–7.0 and SSP5–8.5 scenarios, results indicate that rising temperatures and the corresponding increase in PET are the principal mechanisms driving drought intensification. In contrast, precipitation plays a secondary, more spatially variable role, with smaller effects on SPEI than PET. Under the SSP1–2.6 scenario, the impact of PET and precipitation is more balanced, suggesting that strong mitigation could partially limit temperature-driven drought amplification.

The spatial distribution of the PET effect (Fig. S15) shows a coherent, widespread drying pattern across the entire country, intensifying from SSP1–2.6 to SSP5–8.5. The strongest PET-driven declines in SPEI occur over the central plateau and southern regions, which are already characterized by high aridity and limited moisture availability. In contrast, the precipitation effect (Fig. S16) shows weak, regionally variable signals, with minor wetting tendencies in southeastern and parts of central Iran. This spatial contrast indicates that projected increases in precipitation in some regions are generally insufficient to offset the stronger drying effect caused by increased PET.

Overall, these results indicate that the projected intensification of droughts in Iran is primarily driven by temperature changes rather than precipitation limitations. This finding also explains why meteorological drought hazard can increase under scenarios such as SSP3–7.0, even where precipitation shows slight increases: the hydrological benefit of additional rainfall is outweighed by temperature-driven increases in PET. Our findings are also consistent with those of Salehnia et al.^91^, who used RegCM4 dynamical downscaling to project future changes in PET and SPEI across northeastern Iran. They reported increasing temperature and PET and a progressive shift toward more severe drought conditions by the late century, further supporting the important role of warming-enhanced evaporative demand in future drought intensification in Iran.

To better translate the spatially heterogeneous drought projections into policy-relevant information, Table 2 summarizes the main drought-hazard characteristics for three key regions: the northwest, the central plateau, and the southeast. These regions were selected because they represent distinct drought mechanisms in the projections. The northwest and southeast show relatively high drought frequency and duration in the SPI-based results, whereas the central plateau shows particularly strong PET-driven declines in SPEI. This regional synthesis highlights that future drought conditions in Iran cannot be captured by a single national pattern; rather, different regions are affected by distinct combinations of precipitation variability and warming-driven evaporative demand.

**Table 2:** Regional patterns, climatic drivers, and implications of projected drought hazards in Iran.

Region	Main projected drought signal	Dominant climatic driver	Most relevant indicator	Practical interpretation
Northwest Iran	Higher drought frequency and duration, especially under high-emission scenarios	Reduced or more variable precipitation, together with increasing PET	SPI and SPEI	Drought hazard is linked to both precipitation deficits and warming-driven evaporative demand; monitoring should consider both rainfall anomalies and PET-driven stress
Central plateau	Strongest PET-driven SPEI declines, even where precipitation changes are small or locally positive	Increasing temperature and PET dominate over precipitation changes	SPEI	Water stress may intensify even without large declines in precipitation; this region is especially sensitive to warming-driven increases in atmospheric water demand
Southeast Iran	High drought frequency and prolonged drought hazard, particularly under SSP3–7.0 and SSP5–8.5	A combination of high baseline aridity, precipitation variability, and increasing PET	SPI and SPEI	Drought hazard reflects both rainfall variability and strong evaporative demand; episodic precipitation increases may not fully reduce drought stress

## Discussion

This study presents a comprehensive assessment of future drought characteristics in Iran, using the high-resolution, bias-corrected/downscaled NEX-GDDP-CMIP6 datasets. Two widely recognized drought indices, SPI and SPEI, were applied under four climate change scenarios (SSP1–2.6, SSP2–4.5, SSP3–7.0, and SSP5–8.5). Given the limited availability of high-resolution datasets for the study area, the employed models are based on one of the most widely used and reliable datasets (NEX-GDDP-CMIP6) for climate studies^18,21,32,92–95^. The results for the temperature projection by multi-model means of GCMs show substantial increases in temperature over Iran by the end of the twenty-first century. Based on this study’s results, the temperature in Iran is expected to increase by 1–5 °C in 2071–2100 under SSP scenarios. These findings are consistent with the results presented in previous studies. For example, Behzadi et al.^25^, by investigating several GCMs from CMIP6 models, concluded that the average minimum temperature in 2015–2100 will increase by 4.85 °C, whereas the maximum temperature will increase by 4.9 °C under SSP scenarios. Rahimi et al.^96^ assessed the impacts of climate change in Iran based on 17 GCMs from CMIP5 models under the RCP4.5 and RCP8.5 scenarios. The authors concluded that the annual mean temperature in Iran will increase by 1.9 °C and 3.4 °C by 2041–2070 under RCP4.5 and RCP8.5 scenarios, respectively. The increase in annual temperature for the period 2071–2100 is expected to reach 2.6 °C and 4.1 °C under RCP4.5 and RCP8.5 scenarios, respectively.

Another interesting result shows that based on the spatial distribution (Fig. 6), the increase in temperature in northwest Iran (Urmia Lake basin) is more noticeable than in other regions, as also revealed by Fathian et al.^97^ and Najafi et al.^98^.

The spatial distribution of precipitation in Iran based on the employed models showed that for the projected period (2071–2100), both increasing and decreasing patterns are expected under the SSP scenarios. The projection of precipitation changes in Iran is shown in various estimations in the literature. However, it is essential to highlight that studies that employed CMIP6 models, including our research, generally revealed an increase in precipitation for future periods in Iran under several SSP scenarios^24,25,34,99,100^, which contrasts with prior projections from CMIP5^10,101,102^. The differences in the results are partly related to the selection of model(s), the period of projection, the bias-corrected/downscaled methods, and the selection of scenarios. Although on a global scale, many studies showed that results derived from CMIP6 models exhibit improved and increased projections of future precipitation^103–105^, the uncertainties and biases in projections under climate change scenarios (RCP/SSP) are still challenging^106–109^.

The heterogeneous precipitation projections across Iran under the SSP scenarios, including drying in the northwest and localized wetting in the central, southern, and southeastern regions, can be interpreted in terms of spatially variable changes in the large-scale atmospheric circulation systems that control Iran’s hydroclimate^110^. Western and northwestern Iran receive much of their cold-season precipitation from Mediterranean storm tracks and mid-latitude westerlies. Under global warming, changes in the position and strength of the westerly jet and the Mediterranean circulation may lead to reduced precipitation in these regions^111,112^. These circulation effects are further modulated by Iran’s complex topography, particularly the Zagros and Alborz mountain ranges, which enhance windward precipitation through orographic uplift while contributing to rain-shadow drying across the central plateau^35^. In contrast, central, southern, and southeastern Iran can periodically receive moisture from southern and regional moisture sources, including the Arabian Sea, the Gulf of Oman/Indian Ocean sector, the Persian Gulf, and the Caspian Sea, depending on season and circulation conditions^113^. The localized precipitation increases projected under SSP3–7.0 may therefore be associated with the combined effects of enhanced atmospheric moisture availability and episodic regional moisture transport. Under warmer conditions, the atmosphere can hold more water vapor according to the Clausius–Clapeyron relationship, increasing the potential for higher precipitation when moisture convergence and uplift are present. Therefore, the SSP3–7.0 precipitation signal should be interpreted as a localized and seasonally dependent response rather than a uniform wetting trend across the country^41,114,115^. However, the projected precipitation increases under SSP3–7.0 do not necessarily imply reduced drought hazard. Additional rainfall may occur episodically or as more intense events rather than as a sustained improvement in effective water availability. At the same time, warming-driven increases in PET can offset or exceed the hydrological benefit of increased precipitation. This explains the apparent contrast between lower SPI-based drought probability in some regions and stronger SPEI-based drought conditions under SSP3–7.0. Thus, the SSP3–7.0 results suggest that increased precipitation and increased drought hazard can occur simultaneously when enhanced evaporative demand reduces the effective contribution of additional rainfall.

An increase in the drought events has been reported in many studies for entire regions of Iran or case studies^24,25,102,116–118^. Assessment of drought occurrences in SPI under SSP scenarios revealed that the frequency of droughts for 2071–2100 is relatively high in the northwest and southeast of Iran compared to other regions (Figs. S6–S10 and 7), as shown in several studies^21,119–121^. Moreover, evaluation of drought conditions in SPEI and SSP scenarios showed that in all timescales (i.e., SPEI-1, SPEI-6, and SPEI-12), most regions in Iran experience highly frequent and prolonged droughts, excluding the SSP1–2.6 scenario. The SPEI results emphasized the profound impact of temperature-driven increase in PET, which exacerbates drought severity, particularly in long-term projections under high-emission scenarios (i.e., SSP3–7.0 and SSP5–8.5 scenarios).

Previous studies based on earlier climate models, including CMIP3 and CMIP5, also projected an increasing drought hazard for Iran and the broader Middle East, but there are slight differences in terms of magnitude and regional extent. Studies such as those by Tabari and Willems^122^ and Vaghefi et al.^10^ reported more frequent and prolonged droughts across western and southern Iran by the end of the century, whereas the northern regions displayed weaker or seasonally mixed changes. Regional assessments using CMIP5 simulations^18,123^ generally supported this pattern but revealed variable spatial gradients depending on model selection and climate change scenario. Some CMIP3-based studies even suggested slight precipitation increases in northern Iran^16^, leading to partial wetting trends under specific scenarios.

At the same time, recent multi-decadal studies focusing on the observed changes in droughts in Iran^11,124–127^ consistently show that droughts have intensified in both frequency and severity, accompanied by clear shifts in their spatial distribution. Since the late 1990s, the country has experienced moderate to severe drought conditions almost every year, indicating a significant shift toward more persistent national-scale drought stress. Droughts have become increasingly concentrated in the western, northwestern, and northern regions, with the 1999–2002 period identified as one of the most widespread and intense drought conditions recorded nationwide. At the same time, moderate droughts remain more frequent in the northern and eastern regions, although recent evidence reveals growing penetration of severe droughts into high-elevation and previously less-affected basins. These changes in drought frequency and intensity are strongly associated with increasing temperatures, shifts in precipitation trends, and enhanced evaporative demand, which together have produced negative trends in drought indices across most of Iran, intensifying the long-term persistence of drought conditions.

The results of the present study, based on the employed NEX-GDDP-CMIP6 models, are generally consistent with the direction of these earlier projections but indicate a more spatially coherent and thermodynamically amplified drying signal. This enhancement is mainly due to substantial warming and higher PET simulated by CMIP6 models. Using the high-resolution, bias-corrected NEX-GDDP dataset, the current study resolves finer-scale drought variability, showing that even regions with small precipitation increases experience intensified drought under SPEI due to elevated PET. Thus, while earlier CMIP5/CMIP3 studies captured the onset of regional drying, the CMIP6 ensemble highlights a clear shift toward persistent, temperature-driven droughts, particularly under the SSP3–7.0 and SSP5–8.5 scenarios.

The drought projections in our study align closely with global CMIP6-based assessments, which consistently report intensified and prolonged droughts across arid and semi-arid regions under high-emission scenarios. Li et al.^28^ demonstrated that under SSP2–4.5 and SSP5–8.5 scenarios, approximately 68% of the global land surface will experience intensified drought, with the Middle East identified as one of the most vulnerable regions due to significant variations in precipitation and increased PET. Similarly, Ukkola et al.^128^ found robust future increases in meteorological drought frequency and severity across subtropical belts, including North Africa, Central Asia, and the Middle East, despite uncertainties in precipitation projections. Together, these findings confirm that the projected drying trend in Iran is part of a broader, warming-driven global trend of increasing aridity.

It is worth noting that in drought analysis, selecting a PET estimation method is crucial, as it directly affects the magnitude and variability of temperature-sensitive drought indices such as SPEI. Although the Penman–Monteith method is generally regarded as the physically based reference approach, it requires additional radiative and aerodynamic variables, including radiation, humidity, vapor pressure deficit, and wind speed. In contrast, the Hargreaves–Samani method estimates PET using maximum and minimum temperature together with extraterrestrial radiation, making it practical for large-scale multi-model drought assessments where consistent long-term meteorological inputs are limited.

Omitting wind speed and humidity can introduce region-dependent differences between Hargreaves–Samani and Penman–Monteith-derived PET. In hot, dry, and windy regions, the Hargreaves–Samani equation may underestimate PET relative to the Penman–Monteith equation because it does not explicitly account for the aerodynamic enhancement associated with high wind speed and vapor pressure deficit. In such areas, SPEI values may be less negative than those derived from the Penman–Monteith method, potentially leading to conservative estimates of drought severity. Conversely, in more humid, cloudy, or low-wind regions, the Hargreaves–Samani equation may overestimate PET because the temperature range alone may not fully capture the limiting effects of humidity, cloudiness, and reduced aerodynamic demand. Therefore, the potential bias in SPEI is spatially variable and should be considered when interpreting regional drought patterns. Despite this limitation, several studies support the use of Hargreaves–Samani as a practical PET method for arid and semi-arid drought applications. Raziei and Pereira^65^ evaluated Hargreaves–Samani at 40 Iranian stations and found that it can provide accurate ETo estimates across Iran’s climatic regions. Heydari and Heydari^129^ also showed that calibrated Hargreaves–Samani estimates can achieve acceptable accuracy in semi-arid and arid regions of central Iran. More recently, Saharwardi et al.^67^ and Saharwardi et al.^66^ identified Hargreaves-based PET as suitable for SPEI projections over arid and semi-arid regions in the Middle East (Arabian Peninsula). Lee et al.^130^ found that Hargreaves-based SPEI showed good agreement with Penman–Monteith-based SPEI, particularly at longer accumulation periods. Therefore, in the present study, Hargreaves–Samani-based SPEI is interpreted as a consistent temperature-sensitive drought indicator for comparing relative changes across models, scenarios, and time periods. Although a full PET-method sensitivity assessment using Hargreaves–Samani, Penman–Monteith, Thornthwaite, and other PET formulations would further strengthen the robustness of SPEI-based drought projections, such an analysis requires complete, harmonized, and quality-controlled radiative and aerodynamic variables across all selected GCMs, SSP scenarios, and time periods. Therefore, this comparison is recommended as an important direction for future research.

An additional methodological limitation concerns the stationarity assumption implicit in conventional SPI and SPEI calculations. Both indices are standardized using fitted probability distributions; therefore, their interpretation depends on the statistical properties of the underlying precipitation or climatic water-balance series. Under strong climate change, these properties may evolve over time, including shifts in the mean state, variance, and distribution tails. As a result, drought thresholds based on stationary probability distributions may be affected by quantile drift, in which the same SPI or SPEI value can correspond to different physical levels of water stress under future climatic conditions. This issue is particularly relevant for long-term projections because future precipitation variability and PET may not follow the same distributional behavior as the historical reference climate. Therefore, the SPI and SPEI results presented here should be interpreted as relative changes within a consistent standardized-index framework rather than as absolute drought estimates under fully stationary conditions. Previous studies have emphasized that stationarity assumptions can bias drought assessment under climate change and that drought monitoring frameworks should increasingly account for nonstationary hydroclimatic baselines^131,132^. Future research could extend the present analysis using nonstationary drought frameworks, such as time-varying probability distributions, moving-window standardization, or quantile-based approaches, to evaluate how evolving hydroclimatic distributions may affect projected drought severity and frequency.

The projections presented in this study are based on the ensemble mean of 25 CMIP6 models, which provides a more balanced and credible estimate of future climate conditions than any single model. Since no individual GCM can fully capture the complexity of regional climate dynamics, combining multiple models helps to reduce structural biases and random errors inherent in each simulation^133,134^. The spread among models, represented by the inter-model standard deviation (Fig. 4), reflects differences in various models. Such variability highlights the uncertainty range within the ensemble and indicates that some models may project wetter or drier conditions than the ensemble average. By integrating results across models, the ensemble mean minimizes the influence of outliers and provides a more stable projection for regional-scale drought assessment and adaptation planning.

The projected drought changes should be interpreted within a broader uncertainty framework. Uncertainty enters the analysis through several stages, including emissions scenarios, GCM structure, internal climate variability, statistical downscaling and bias correction, PET estimation, drought-index selection, and the stationarity assumptions used in SPI/SPEI calculation. Scenario uncertainty reflects the different future forcing pathways represented by the SSPs, while GCM structural uncertainty arises from differences in how individual models simulate atmospheric circulation, temperature, precipitation, and land–atmosphere interactions. Internal climate variability further affects precipitation and drought variability, particularly at regional scales. Although NEX-GDDP-CMIP6 provides bias-corrected and downscaled climate projections and generally improves precipitation representation relative to raw GCM outputs, uncertainty may still remain for localized high-precipitation events, especially in regions influenced by complex topography, orographic precipitation, and strong spatial rainfall variability. This uncertainty is particularly relevant for SPI-based drought projections because SPI is derived solely from precipitation. Therefore, SPI results are interpreted here as regional-scale indicators of precipitation-driven drought change, while SPEI provides additional information on warming-driven evaporative demand. Consequently, the combined use of SPI and SPEI provides a more comprehensive assessment of future drought conditions than either index alone, while the results should be interpreted as plausible ranges of future drought conditions rather than deterministic predictions.

In this study, these uncertainties were partly addressed by using a 25-model ensemble, four SSP scenarios, 30-year analysis periods, inter-model uncertainty ranges, historical model evaluation, and a combined SPI/SPEI framework. The inter-model ranges show that temperature projections are more robust across models, whereas precipitation projections contain larger uncertainty. Therefore, the projected drought changes should be interpreted as plausible ranges of future drought conditions rather than deterministic predictions. Findings that are consistent across scenarios and indices, such as widespread warming and PET-driven SPEI intensification, can be considered more robust, while the exact magnitude and spatial distribution of precipitation-driven SPI changes should be interpreted with greater caution.

While projections generally indicate substantial warming, changes in extreme precipitation, and severe, prolonged drought events, the magnitude and reliability of these changes depend on climate models and geographical regions. This variability, particularly in projections of extreme events such as droughts and floods, underscores the need for adaptable planning strategies that consider a wide range of possible future conditions. Thus, the results of this study have significant implications for managers and policymakers in Iran, supporting proactive measures to build climate resilience and adaptation.

The seasonal pattern of SPEI drought probability has important implications for both rain-fed and irrigated agriculture in Iran. Higher SPEI drought probability in spring and summer is particularly critical because these seasons overlap with key crop-growth stages, increasing temperature, and peak atmospheric evaporative demand. In rain-fed agriculture, spring droughts can reduce soil moisture during germination, vegetative growth, and grain filling, increasing the risk of yield loss. This is especially important in regions where crop production depends strongly on winter and spring precipitation. In irrigated agriculture, higher summer SPEI drought probability indicates increased crop water requirements and higher irrigation demand, which can intensify pressure on surface water allocations, reservoirs, and groundwater resources.

The increasing persistence of drought conditions during autumn and winter also has important implications. Persistent winter/autumn SPEI droughts may reduce soil moisture recharge before the growing season and limit the carryover water available for spring crops. This weakens the natural buffering capacity of rain-fed systems and can increase early-season irrigation demand in irrigated systems. Therefore, the seasonal SPEI results suggest that future agricultural drought conditions in Iran may unfold as a sequence of reduced cold-season recharge, followed by intensified spring and summer evaporative demand. These seasonal changes define three practical adaptation windows. During autumn and winter, when soil moisture, reservoir, and groundwater recharge normally support water availability for the following growing season, persistent SPEI deficits indicate the need for early assessment of reservoir carryover storage, aquifer recharge opportunities, and preliminary water allocations. During spring crop establishment and development, increased drought probability supports intensified soil-moisture monitoring, flexible planting calendars, and the selection of drought-tolerant crops, particularly in rain-fed systems. During the summer peak-demand period, increasing PET supports climate-informed irrigation scheduling, reservoir-release planning, and groundwater-allocation limits based on projected rather than historical evaporative demand. The implementation of these measures should be tailored to local crop calendars, basin-level water availability, and irrigation practices.

Iran’s exposure to persistent drought and its heavy dependence on groundwater make the projected changes in SPI and SPEI particularly critical for long-term water and food security. The projected increase in drought frequency and duration will exacerbate existing stresses on agriculture, ecosystems, and water infrastructure. In particular, the projected long-duration SPEI droughts, including drought episodes exceeding 20 months under SSP3–7.0 and SSP5–8.5, suggest that moisture deficits may persist across multiple seasons or successive hydrological years. Such prolonged droughts could reduce the reliability of surface water allocation, increase crop water stress, and intensify reliance on groundwater for irrigation in major agricultural basins. This is especially relevant for central Iran, including the Zayandeh-Rud basin, where drought conditions have historically reduced surface-water availability and increased groundwater substitution in agriculture^135^. In the northwest, prolonged droughts may further increase pressure on the Lake Urmia basin, where climatic variability and agricultural water extraction strongly affect lake-water availability and ecosystem stability^136^. For urban water supplies, multi-season drought persistence can reduce reservoir recharge and increase pressure on groundwater reserves, particularly in densely populated basins such as the Salt Lake basin, which includes major urban centers such as Tehran and Qom. These findings underscore the need for a shift from short-term crisis management to resilience-oriented adaptation. In the northwestern and western provinces, where drying trends dominate, improving irrigation efficiency, adjusting cropping calendars, and investing in managed aquifer recharge would help stabilize agricultural productivity. The central plateau, characterized by chronic groundwater decline, requires stricter regulation of water abstraction, the large-scale reuse of wastewater, and a gradual adoption of alternative water sources, such as desalination. Even in the regions where precipitation may increase but evaporation remains high, small- and medium-scale storage systems, watershed rehabilitation, and reforestation could help capture episodic rainfall and mitigate land degradation. At the national scale, developing climate-informed drought early warning systems and integrating water, agricultural, and energy planning are crucial for strengthening coordination and preparedness. These regionally tailored measures translate the projected drought risks into actionable strategies that promote sustainable resource management and long-term resilience under a warming climate.

As a notable highlight, it is worth mentioning that while SPI and SPEI are widely recognized for assessing meteorological drought, they primarily capture climate-driven variability in precipitation and PET. However, anthropogenic drivers (e.g., intensive groundwater withdrawal, irrigation expansion, mismanagement, deforestation, and urbanization) can exacerbate drought severity by altering land–atmosphere interactions and water availability^137–139^. In Iran, where groundwater accounts for more than half of total water consumption, extensive pumping has lowered water tables and reduced soil moisture resilience, thereby intensifying the impacts of agricultural and hydrological droughts even during normal precipitation years^140,141^. In addition, declining groundwater recharge diminishes effective subsurface water storage and weakens resilience to prolonged drought^142^. Consequently, the projected increase in meteorological drought frequency under future climate scenarios should be interpreted as a conservative estimate, since human-driven water and land management pressures are likely to further intensify total drought risk. Addressing these compound effects will require coupling climate projections with socio-hydrological models and sustainable water policy frameworks to develop realistic adaptation plans.

## Conclusion

This research, to our knowledge, is one of the most comprehensive assessments of future drought conditions in Iran, based on a high-resolution and bias-corrected NEX-GDDP-CMIP6 dataset under SSP scenarios. The novelty of this study lies in combining an Iran-specific evaluation of the NEX-GDDP-CMIP6 ensemble with a multi-timescale SPI–SPEI comparison, a process-based interpretation of projected climate changes, and a quantitative attribution of future drought changes to precipitation and PET. The results indicate an increase in temperature in Iran under all SSP scenarios, with the most significant increase under high-emission scenarios (SSP3–7.0 and SSP5–8.5), where temperature is expected to rise by 4–5 °C in most parts of the country. Precipitation projections reveal slight variability, with some regions experiencing slight increases, particularly under SSP3–7.0, where precipitation is expected to increase by up to 40% in central, southern, and eastern regions. Drought frequency and duration are projected to increase, particularly in the northwest and southeast regions. Assessment of drought characteristics in SPEI and SSP scenarios showed that in all timescales (i.e., SPEI-1, SPEI-6, and SPEI-12), most regions in Iran experience highly frequent and prolonged droughts, excluding the SSP1–2.6 scenario, which highlights the role of temperature-driven increases in PET in intensifying drought hazard. Because the 0.25° NEX-GDDP-CMIP6 grid smooths sharp orographic gradients, projected drought-hazard intensities at the highest elevations of the Alborz and Zagros ranges carry greater localized uncertainty and may not fully capture station-scale precipitation and drought extremes. These findings emphasize the growing risk of water stress and the need for robust water resource management strategies in Iran. In addition, this study provides valuable insights to policymakers and stakeholders in prioritizing climate adaptation measures to enhance resilience against future droughts, particularly in vulnerable regions.

## Supplementary Information

Below is the link to the electronic supplementary material.

Supplementary Material 1

## Data Availability

All datasets of high-resolution NEX-GDDP-CMIP6 used for this study are publicly available at https://registry.opendata.aws/nex-gddp-cmip6 (accessed in December 2023). The additional data and codes supporting this study’s findings are available at https://zenodo.org/records/17834101. All packages and tools used in this research are publicly available. The PET, SPI, and SPEI values have been calculated with the Climate Indices Python package (https://github.com/monocongo/climate_indices; version 1.0.10). Additionally, the postprocessing of the SPI, SPEI, and Climate data results has been carried out with the Climate Data Operators (CDO: https://code.mpimet.mpg.de/projects/cdo) and the corresponding Python scripts for visualizing the results.
