# Supplementary material for: Assessment of future meteorological droughts in Iran
Source: Sci Rep. 2026 Aug 28;16:27080. doi: 10.1038/s41598-026-65752-6 (PMC13524922; doi:10.1038/s41598-026-65752-6)
Supplement: Supplementary file 1 [file 41598_2026_65752_MOESM1_ESM.docx]

Supplementary Material for “**Assessment of Future Meteorological Droughts in Iran”**

Babak Ghazi ^1^*, Hossein Salehi ^2*^, Marco Turco ^3^, Amir AghaKouchak ^4,5,6 *^

^1^ Department of Geoscience, University of Nevada, Las Vegas, United States of America

^2^ Department of Physics, University of Trento, Trento, Italy

^3^ Regional Atmospheric Modelling Group, Regional Campus of International Excellence Campus Mare Nostrum, University of Murcia, Murcia 30100, Spain

^4^ Department of Civil and Environmental Engineering, University of California, Irvine, CA, USA

^5^ Department of Earth System Science, University of California, Irvine, CA, USA

^6^ United Nations University Institute for Water, Environment and Health, Hamilton, ON, Canada

*Corresponding authors: Babak Ghazi, [ghazi@unlv.nevada.edu](mailto:ghazi@unlv.nevada.edu), Hossein Salehi, [hossein.salehi@unitn.it](mailto:hossein.salehi@unitn.it), Amir AghaKouchak, [amir.a@uci.edu](mailto:amir.a@uci.edu).

**Contents of this file**

Table S1 to S3

Calculation of PET, SPI, and SPEI

Figures S1 to S16

Table S1. List of NEX-GDDP-CMIP6 GCM models employed in this study

| **No.** | **Model** | **No.** | **Model** |
| --- | --- | --- | --- |
| 01 | ACCESS-CM2 | 14 | INM-CM4-8 |
| 02 | ACCESS-ESM1-5 | 15 | INM-CM5-0 |
| 03 | BCC-CSM2-MR | 16 | IPSL-CM6A-LR |
| 04 | CanESM5 | 17 | KACE-1-0-G |
| 05 | CESM2 | 18 | MIROC6 |
| 06 | CMCC-CM2-SR5 | 19 | MPI-ESM1-2-HR |
| 07 | CMCC-ESM2 | 20 | MPI-ESM1-2-LR |
| 08 | CNRM-CM6-1 | 21 | MRI-ESM2-0 |
| 09 | CNRM-ESM2-1 | 22 | NorESM2-LM |
| 10 | EC-Earth3 | 23 | NorESM2-MM |
| 11 | FGOALS-g3 | 24 | TaiESM1 |
| 12 | GFDL-ESM4 | 25 | UKESM1-0-LL |
| 13 | GISS-E2-1-G |  | |

Table S2. Geographic location and characteristics of selected synoptic stations in Iran

| **No.** | **Station name** | **Latitude** | **Longitude** | **No.** | **Station name** | **Latitude** | **Longitude** |
| --- | --- | --- | --- | --- | --- | --- | --- |
| 01 | Abadan | 30.38 | 48.21 | 27 | Maku | 39.38 | 44.39 |
| 02 | Abadeh | 31.20 | 52.62 | 28 | Maragheh | 37.35 | 46.15 |
| 03 | Abali | 35.75 | 51.88 | 29 | Mashhad | 36.24 | 59.63 |
| 04 | Abumusa Island | 25.87 | 55.01 | 30 | Masjedsoleyman | 31.98 | 49.24 |
| 05 | Ahvaz | 31.34 | 48.74 | 31 | Minab | 27.11 | 57.09 |
| 06 | Ardebil | 38.22 | 48.33 | 32 | Omidiyeh (Aghajari) | 30.74 | 49.69 |
| 07 | Bandar Abbas | 27.21 | 56.37 | 33 | Orumiyeh | 37.66 | 45.06 |
| 08 | Bandar-E-Lengeh | 26.53 | 54.83 | 34 | Parsabad | 39.60 | 47.78 |
| 09 | Birjand | 32.89 | 59.28 | 35 | Qazvin | 35.70 | 50.06 |
| 10 | Bojnurd | 37.49 | 57.30 | 36 | Quchan | 37.12 | 58.45 |
| 11 | Chabahar | 25.28 | 60.65 | 37 | Sabzevar | 36.21 | 57.65 |
| 12 | Esfahan | 32.52 | 51.71 | 38 | Sarakhs | 36.54 | 61.15 |
| 13 | Esfahan (Airport) | 32.74 | 51.86 | 39 | Semnan | 35.59 | 53.42 |
| 14 | Fasa | 28.90 | 53.72 | 40 | Shahrud | 36.42 | 55.09 |
| 15 | Ferdows | 34.03 | 58.18 | 41 | Shiraz | 29.56 | 52.60 |
| 16 | Hamedan (Airport) | 34.87 | 48.53 | 42 | Sirjan Airport | 29.47 | 55.68 |
| 17 | Iranshahr | 27.23 | 60.72 | 43 | Sirjan City | 29.55 | 55.66 |
| 18 | Jask | 25.64 | 57.77 | 44 | Tabas | 33.60 | 56.95 |
| 19 | Jolfa | 38.93 | 45.60 | 45 | Tabriz | 38.12 | 46.24 |
| 20 | Karaj | 35.81 | 50.95 | 46 | Tehran (Airport) | 35.69 | 51.31 |
| 21 | Kashan | 33.97 | 51.48 | 47 | Torbat-E Heydariyeh | 35.33 | 59.21 |
| 22 | Kerman | 30.26 | 56.96 | 48 | Yazd | 31.90 | 54.29 |
| 23 | Kermanshah | 34.35 | 47.15 | 49 | Zabol | 31.09 | 61.54 |
| 24 | Khoy | 38.56 | 45.00 | 50 | Zahedan | 29.47 | 60.90 |
| 25 | Kish Island | 26.52 | 53.98 | 51 | Zanjan | 36.66 | 48.52 |
| 26 | Mahabad | 36.75 | 45.72 |  |  |  |  |

Table S3. Drought Parameter (frequency and duration) Equations

| **Drought parameter** | **Equation** | **Symbol description** |
| --- | --- | --- |
| Frequency (Probability) |   $F= \frac{n_{m}}{N} \times100$ (1) | n_m_: number of drought months  N: total number of months in the historical or projected timeframe being evaluated. |
| Duration |    (2) | X_t_: SPI/SPEI value at time t  T: drought threshold value  1(X_t_<T) is an indicator function that assigns 1 when X_t_<T and 0 otherwise  ti, tj = the termination and onset time points, respectively, of an individual drought event  max selects the longest drought duration |

**The detailed procedure for calculation of PET, SPI, and SPEI.**

The SPI/SPEI are computed using Pearson Type III distributions. The calculation of PET was estimated based on the Hargreaves and Samani equation (Hargreaves & Samani, 1985), as one of the most applied empirical approaches for estimating PET. It relies on minimum and maximum temperatures and extraterrestrial radiation. The equation for calculating PET, based on the Hargreaves and Samani method is described as follows:

$PET=0.0023R_{a}\left(\frac{T_{max}+T_{min}}{2}+17.8\right)\sqrt{T_{max}-T_{min}}$ (3)

Where *Tmin* and *Tmax* are the minimum and maximum temperatures, respectively, and *Ra* is the extraterrestrial radiation.

In the calculator of SPI utilizing Pearson Type III distribution**,** the probability density function (PDF) of the Pearson Type III distribution is calculated based on Eq. (4):

$$f(x)=\frac{\left( x-\alpha\right)^{\beta-1}ⅇ^{-\frac{x-\alpha}{\gamma}}}{\gamma^{\beta}\Gamma\left( \beta\right)} (4)$$

Where *x* is precipitation amount, *α* is the location parameter (often set to 0 for precipitation data), *β* is the shape parameter, *γ* is the scale parameter, and *Γ(β)* is the gamma function.

Once the Pearson Type III distribution is fitted to the precipitation data, the cumulative probability *F(x)* for a given precipitation amount x is obtained using Eq. (5):

$$F(x)=P(X\leq x)=\int_{0}^{x} f\left( t \right)ⅆt (5)$$

The SPI is then derived (Eq. 6) by transforming the cumulative probability *F(x)* into a standard normal variable using the inverse standard normal function (Φ−1):

$$SPI=\Phi^{-1}\left( F\left( x \right) \right) (6)$$

Where *Φ−1* is the inverse of the standard normal cumulative distribution function (CDF).

Since the Pearson Type III distribution does not have a direct analytical transformation to a standard normal distribution, an approximation is often used to calculate SPI (Eq. 7)

$$SPI=\frac{x-\mu}{\sigma} (7)$$

Where *x* is precipitation, *μ* is the mean of the fitted Pearson Type III distribution, and σ is the standard deviation of the fitted distribution.

Calculating SPEI follows a similar approach to SPI but is applied to the climatic water balance (WB) rather than only to precipitation. To compute the SPEI value using the Pearson Type III distribution, the water balance (WB) difference is normalized. The probability density function can be expressed using the following equation (Eq. 8):

$$f(x)=\frac{\left( x-\alpha\right)^{\beta-1}ⅇ^{-\frac{x-\alpha}{\gamma}}}{\gamma^{\beta}\Gamma\left( \beta\right)} (8)$$

Here, *x* is the water balance value (WB = P − PET), *α* represents the location parameter, *β* corresponds to the shape parameter, *γ* is the scale parameter, and Γ(β) is the standard gamma function. Thus, the probability distribution function can be formulated as follows (Eq. 9):

$$F(x)=P(X\leq x)=\int_{-\infty}^{x} f\left( t \right)ⅆt (9)$$

SPEI is defined as follows (Eq. 10):

$$SPEI=W-\frac{C_{0}+C_{1}W+C_{2}W^{2}}{1+d_{1}W+d_{2}W^{2}+d_{3}W^{3}} (10)$$

Whereas $W=\sqrt{-2ln\left( P \right)}when P\leq0.5$, and $W=\sqrt{-2ln\left( 1-P \right)} when P>0.5$(where *P* is the probability of exceeding a determined WB value), C_0_ = 2.5155, C_1_ = 0.8028, C_2_ = 0.0203, d_1_ = 1.4327, d_2_ = 0.1892, d_3_ = 0.0013

Fig. S1. Elevation-based comparison of observed and NEX-GDDP-CMIP6 ensemble-mean cumulative distribution functions for monthly precipitation and mean temperature during the historical period (1985–2014). Stations were grouped into lowland (0–500 m), mid-altitude (500–1000 m), high-elevation (1000–2000 m), and Alpine high-mountain (>2000 m).

Fig. S2. Historical variations in the SPI and SPEI calculated at 1-, 6-, and 12-month timescales for meteorological stations across Iran (1985–2014).

Fig. S3. Statistically robust future changes in temperature and precipitation over Iran (Hatching indicates grid cells where projected changes are statistically significant/robust across the GCM ensemble).

Fig. S4. Area-mean future trends in temperature and precipitation over Iran (Box plots show GCM-level Sen’s slopes; symbols indicate trend direction, and shading indicates Mann–Kendall significance).

Fig. S5. Monthly climatology of mean temperature (a) and precipitation (b) over Iran during the historical period (1985–2014) for the ensemble mean and the 25 individual NEX-GDDP-CMIP6 models, illustrating the seasonal structure of inter-model spread.

Fig. S6. Spatial distribution in frequency (probability) of droughts based on SPI-01 and SPEI-01 under SSP scenarios in the projected period (2071–2100)

Fig. S7. Spatial distribution in frequency (probability) of droughts based on SPI-06 and SPEI-06 under SSP scenarios in the projected period (2071–2100)

Fig. S8. Spatial distribution in the duration of droughts based on SPI-01 and SPEI-01 under SSP scenarios in the projected period (2071–2100)

Fig. S9. Spatial distribution in the duration of droughts based on SPI-06 and SPEI-06 under SSP scenarios in the projected period (2071–2100)

Fig. S10. Spatial Variability of drought duration in Iran as estimated by SPI-12 and SPEI-12 for the future period (2071–2100) under SSP scenarios.

Fig. S11. Spatial change in the duration of extreme droughts (−2.0 ≥ SPI/SPEI) in Iran under SSP scenarios in the projected period (2071–2100)

Fig. S12. Drought probability (top) and duration (bottom) variations estimated by SPI and SPEI under SSP scenarios in Iran for the projected period (2041–2070).

Fig. S13. Seasonal drought probability across Iran for the period 2071–2100 based on SPI and SPEI at 1-, 6-, and 12-month scales under four SSP scenarios (SSP1-2.6, SSP2-4.5, SSP3-7.0, and SSP5-8.5).

Fig. S14. Distribution of precipitation and PET attribution on changes in the Standardized Precipitation Evapotranspiration Index (SPEI) across Iran under SSP scenarios from CMIP6 in 2071-2100

Fig. S15. Spatial distribution of the PET effect on changes in the SPEI across Iran under SSP scenarios from CMIP6, and at three timescales (SPEI-1, SPEI-6, and SPEI-12) for the period 2071-2100.

Fig. S16. Spatial distribution of the precipitation effect on changes in the SPEI across Iran under SSP scenarios from CMIP6, and at three timescales (SPEI-1, SPEI-6, and SPEI-12) for the period 2071-2100.

**Reference:**

Hargreaves, G. H., & Samani, Z. A. (1985). Reference crop evapotranspiration from temperature. *Applied engineering in agriculture*, *1*(2), 96-99.
